# Assessment of Trends in Statin Therapy for Secondary Prevention of Atherosclerotic Cardiovascular Disease in US Adults From 2007 to 2016

**DOI:** 10.1001/jamanetworkopen.2020.25505

**Published:** 2020-11-20

**Authors:** Xiaoxi Yao, Nilay D. Shah, Bernard J. Gersh, Francisco Lopez-Jimenez, Peter A. Noseworthy

**Affiliations:** 1 Robert D. and Patricia E. Kern Center for the Science of Health Care Delivery, Mayo Clinic, Rochester, Minnesota; 2 Division of Health Care Policy and Research, Department of Health Sciences Research, Mayo Clinic, Rochester, Minnesota; 3 Department of Cardiovascular Medicine, Mayo Clinic, Rochester, Minnesota; 4 OptumLabs, Cambridge, Massachusetts

## Abstract

**Importance:**

Atherosclerotic cardiovascular disease (ASCVD) is highly prevalent in the US, with studies indicating substantial rates of nonadherence to and undertreatment with statin therapy. The 2013 American College of Cardiology/American Heart Association guideline recommended high-intensity statins for all patients age 75 years and younger with documented ASCVD in whom such therapy is tolerated, but there is limited evidence documenting population trends of statin use, adherence, and outcomes in the periods before and after the update to the guideline.

**Objective:**

To assess trends in the use, adherence, cost, and outcomes of statin therapy for secondary prevention in patients with different types of ASCVD between 2007 and 2016.

**Design, Setting, and Participants:**

This retrospective cohort study used data from the OptumLab Data Warehouse database containing privately insured and Medicare Advantage enrollees with demographic characteristics similar to the national US population. Participants were adult patients (age ≥21 years) who had their first ASCVD event between January 1, 2007, and December 31, 2016. Data were characterized as belonging to 3 groups: (1) cardiovascular heart disease (CHD); (2) ischemic stroke or transient ischemic attack (TIA); and (3) peripheral artery disease (PAD). Data were analyzed from July 1 to August 1, 2018.

**Exposures:**

Calendar year of the initial ASCVD event.

**Main Outcomes and Measures:**

Trends in the statin use (within 30 days of discharge from hospitalization), adherence (proportion of days covered ≥80% within the first year), cost, major adverse cardiac events (1-year cumulative risk), and statin intolerance (within the first year).

**Results:**

Of the 284 954 patients with a new ASCVD event, 128 422 (45.1%) were women; the median age was 63 years (interquartile range [IQR], 54-72 years); 207 781 (72.9%) were White. The use of statins increased from 50.3% in 2007 to 59.9% in 2016, the use of high-intensity statins increased from 25.0% to 49.2%, and the adherence increased from 58.7% to 70.5% (*P* < .001 for all trends). Patients with CHD were more likely to receive statins and high-intensity statins and adhere to medications than patients with ischemic stroke, TIA, or PAD despite similar observed treatment benefit. In 2016, 80.9% of patients with CHD used a statin vs 65.8% of patients with ischemic stroke or TIA and 37.5% of patients with PAD. Out-of-pocket cost per 30-day decreased from a median of $20 (interquartile range, $7.6-$31.9) in 2007 to $2 (interquartile range, $1.6-$10.0) in 2016 (*P* < .001) with the increasing use of generic statins (42.0% in 2007 vs 94.9% in 2016; *P* < .001). Major adverse cardiac events decreased from 8.9% in 2007 to 6.5% in 2016 (*P* < .001) whereas statin intolerance increased from 4.0% to 5.1% (*P* < .001).

**Conclusions and Relevance:**

There have been modest improvements in the use, adherence, and cardiovascular outcomes over the past decade for statin therapy in patients with ASCVD, but a substantial and persistent treatment gap exists between patients with and without CHD, between men and women.

## Key Points

**Question:**

What are the use, adherence rate, cost, and outcome trends in statin therapy for secondary prevention of atherosclerotic cardiovascular disease (ASCVD) in US adults from 2007 to 2016?

**Findings:**

In this cohort study of data from 284 954 patients, modest increases in statin use, statin adherence rates, and cardiovascular outcomes among patients with ASCVD from 2007 to 2016 were found. The use of high-intensity statins approximately doubled over this time frame.

**Meaning:**

Results of this study suggest that the increases in statin use, statin adherence rates, and cardiovascular outcomes were modest; and that substantial and persistent treatment gap exists.

## Teaser

This cohort study assesses 10-year use, adherence, cost, and outcome trends in statin therapy for atherosclerotic cardiovascular disease in US adults using nationwide research center database information.

## Introduction

Atherosclerotic cardiovascular disease (ASCVD) is highly prevalent, affecting approximately 20 million people in the US.^1,2^ Statin therapy is a pillar of secondary prevention for these patients,^3^ but numerous studies showed a substantial rate of nonadherence and undertreatment.^4,5^ The benefits of adhering to statin therapy have been reported.^6,7,8^ The past decade has witnessed extensive efforts and innovative interventions to improve statin adherence, however with varying degrees of success.^9^ The 2013 American College of Cardiology/American Heart Association (ACC/AHA) recommended high-intensity statins for all patients age 75 years and younger with documented ASCVD in whom such therapy is tolerated,^10^ and generic high-intensity statins have become widely available.

However, limited evidence documents population trends of statin use, adherence, and outcomes. Previous studies of statin use focused on patients with myocardial infarction (MI) and use of guideline-recommended statin therapy; few studies have examined trends of statin use in patients with other types of ASCVD, such as ischemic stroke or peripheral artery disease (PAD).^1,11,12^ Furthermore, although an increase in the statin use and intensity could be expected to lead to a decrease in major adverse cardiac events (MACE), few data existed to demonstrate the population trend of MACE in patients with ASCVD.

The current study used a large national cohort of patients with all types of ASCVD managed at diverse routine practice settings to examine the use, adherence, cost, and outcomes of statin therapy for secondary prevention between 2007 and 2016. Specifically, the goal was to assess whether there have been improvements in treatment and outcomes and to identify gaps to guide future quality improvement efforts.

## Methods

The Mayo Clinic Institutional Review Board determined that this study was exempt from review and informed patient consent because the study used preexisting deidentified data. This study followed the Strengthening the Reporting of Observational Studies in Epidemiology (STROBE) reporting guideline for cohort studies.

### Study Population

Adult patients (age ≥21 years) who had their first ASCVD event between January 1, 2007, and December 31, 2016, were identified using the OptumLab Data Warehouse, which contains data from more than 160 million privately insured and Medicare Advantage enrollees of all ages and races from all 50 states managed at diverse practice settings.^13,14^ The population is broadly representative of patients in routine practice. The distribution of age, sex, and race/ethnicity in OptumLabs enrollees is similar to a previous study using a nationally representative cohort from the Medical Expenditure Panel Survey (eTable 1 in the Supplement).^1^ This database has been used in numerous previous studies on treatment trends and outcomes.^15,16,17^ Data were analyzed from July 1 to August 1, 2018.

ASCVD was defined as MI, angina, coronary revascularization, ischemic stroke, transient ischemic attack (TIA), or PAD.^10^ The date of a patient’s first ASCVD event was defined as the index date. A total of 1404 (0.5%) of patients had multiple types of ASCVD events on the same day and were excluded from the study. If a patient was hospitalized, the index date was the discharge date from the hospital. MI, angina, ischemic stroke, and TIA were identified based on the primary diagnosis of an emergency department visit or hospitalization. Patients with angina were required to have additional diagnosis codes indicating the presence of coronary artery disease at baseline or on the index date to avoid the inclusion of patients with noncardiac chest pain. Patients with ischemic stroke or TIA were excluded if they had a diagnosis of atrial fibrillation at baseline or on the index date to limit ischemic stroke or TIA presumed to be of atherosclerotic origin.^18^ Coronary revascularization was identified using procedure codes. Diagnosis codes of PAD alone are often not specific or not sensitive, so PAD was identified using a validated algorithm using a combination of diagnosis and procedure codes with positive predictive value of 91%.^19^

Patients were required to have continuous medical and pharmacy insurance coverage for at least 1 year before the index date to allow sufficient data to characterize the patient’s medical history. Patients were excluded if they died or discontinued health insurance within the 30 days of discharge, had an index hospitalization lasting more than 30 days, received hospice or skilled nursing facility care within 30 days of the index date, or had invalid or missing age and sex data (eFigure 1 in the Supplement). Patient race/ethnicity provided by OptumLabs was classified as non-Hispanic White (White), non-Hispanic Black (Black), Asian, Hispanic, or other/unknown. Self-report was the primary source, and when it was missing, imputation was made through the database based on other available administrative data.^20^ For patient medical history and outcomes, there were no missing data because the variables were defined by the presence of a claim with eligible diagnosis codes, procedure codes, or prescription fills. The absence of such claims was interpreted as the absence of the condition.

### Exposure and Outcomes

The main independent exposure variable was the calendar year of a patient’s initial ASCVD event. The outcomes included statin use, adherence, cost, and clinical outcomes. Patients were followed up until December 31, 2017, or the date of disenrollment from health insurance plans or death, whichever happened first. The use of statin therapy was defined as a prescription fill captured in pharmacy claims within 30 days of discharge. A sensitivity analysis was performed to assess statin use within 90 days of discharge. Among patients who used a statin within 30 days, the use of high-intensity statin was examined, defined as atorvastatin 40 mg or 80 mg, rosuvastatin 20 mg or 40 mg, and simvastatin 80 mg.^10^ Adherence was measured by the proportion of days covered (PDC) at the end of 1 year. Patients with a PDC greater than or equal to 80% were considered as being adherent to statin therapy. When calculating the PDC, all statins were considered regardless of whether the dose was changed or the patient switched from one agent to another. The adherence analysis was limited to patients who used statins within the first 30 days and had continuous enrollment in health insurance for the first 12 months during follow-up. Among patients who used statins within 30 days of discharge, cost per 30-day supply was calculated, adjusted for inflation to reflect the cost in 2016 dollars.^21,22^ The 1-year cumulative risk of MACE was calculated, including MI, ischemic stroke, revascularization, and all-cause mortality. Statin intolerance was assessed during the first year after the ASCVD event, defined using an established algorithm^23,24^ which includes down-titration of statin dose or switch to a lower-intensity statin,^1^ initiation of ezetimibe or a proprotein convertase subtilisin/kexin type 9 inhibitor within 7 days before or any time after or discontinuing statins,^2^ diagnosis for rhabdomyolysis or adverse effect of an antihyperlipidemic agent,^3^ or fills for 3 or more different statins.^4^

### Statistical Analysis

The trends were assessed in the overall population as well as in the 3 subgroups defined based on a patient’s initial ASCVD event^1^: MI, angina, and coronary revascularization (hereafter referred to as coronary heart disease [CHD])^2^; ischemic stroke and TIA^3^; PAD. There were some changes in the patient mix over time because the expansion of Medicare Advantage plans was associated with a greater number of patients age 65 years and older in the database (eTable 2 in the Supplement). Therefore, a weight was applied so the cohorts from 2007 to 2015 were similar to the cohort in 2016 in all analyses. The weight was calculated by a multinomial logistic regression, with the year of the index date as the outcome, and all the patient characteristics in Table 1 as the independent variable. The baseline characteristics were similar across years after applying the weight (eTable 3 in the Supplement). Sensitivity analyses were performed assessing the results with and without weights (eTable 4 in the Supplement). Logistic regression was used to assess the trends of binary outcomes, including statin use, adherence, and statin intolerance. Linear regression was used to assess the trends in costs. Multivariable logistic regression models were used to assess patient characteristics (age, sex, race, ASCVD type, and comorbidities) associated with statin use or adherence. Cox proportional hazards regression models were used to assess MACE, and the cumulative risk of MACE at the end of 1 year was calculated using Kaplan-Meier survivor function. A multivariable Cox proportional hazards regression model was used to assess the association between MACE and statin use, adjusted for age, sex, race, and comorbidities in the overall cohort and in each of the ASCVD subtypes. A 2-sided *P* < .05 was considered to indicate statistical significance.

**Table 1.** Patient Characteristics

Characteristic	No. (%)			
	MI, angina, or revascularization (n = 104 500)	Ischemic stroke or TIA (n = 60 866)	PAD (n = 119 588)	Total (N = 284 954)
Age, median (IQR), y	61 (54-70)	62 (53-73)	64 (54-74)	63 (54-72)
Female sex	31 742 (30.4)	32 026 (52.6)	64 654 (54.1)	128 422 (45.1)
Race				
Asian	2336 (2.2)	1352 (2.2)	4779 (4.0)	8467 (3.0)
Black	9385 (9.0)	8370 (13.8)	16 954 (14.2)	34 709 (12.2)
Hispanic	6587 (6.3)	4606 (7.6)	13 721 (11.5)	24 914 (8.7)
White	82 665 (79.1)	44 769 (73.6)	80 347 (67.2)	207 781 (72.9)
Other/unknown	3527 (3.4)	1769 (2.9)	3787 (3.2)	9083 (3.2)
Hypertension	86 606 (82.9)	48 573 (79.8)	91 874 (76.8)	227 053 (79.7)
Diabetes	34 774 (33.3)	17 759 (29.2)	43 344 (36.2)	95 877 (33.6)
Stage 3-5 CKD	7300 (7.0)	3861 (6.3)	8296 (6.9)	19 457 (6.8)
Heart failure	23 057 (22.1)	5111 (8.4)	11 945 (10.0)	40 113 (14.1)

Abbreviations: CKD, chronic kidney disease; IQR, interquartile range; MI, myocardial infarction; PAD, peripheral artery disease; TIA, transient ischemic attack.

Subgroup analyses were performed by age group (age <65 years, 65-74 years, and ≥75 years) and sex. In patients with PAD, in addition to MACE, the lower extremity vascular complication was examined, which included surgical or endovascular procedures and amputations. The secondary and subgroup analyses (all analyses except the trends of statin use, cost, adherence, and clinical outcomes in the overall population) were considered to be exploratory. Details of the methods can be found in the eAppendix in the Supplement.

## Results

Among 284 954 patients with a new ASCVD event, 104 500 had CHD, 60 866 had ischemic stroke or TIA, and 119 588 had PAD. The median age in the overall cohort was 63 years (interquartile range [IQR], 54-72 years); 128 422 (45.1%) of patients were women and 207 781 (72.9%) were White (Table 1).

### Statin Use

**Figure 1.**
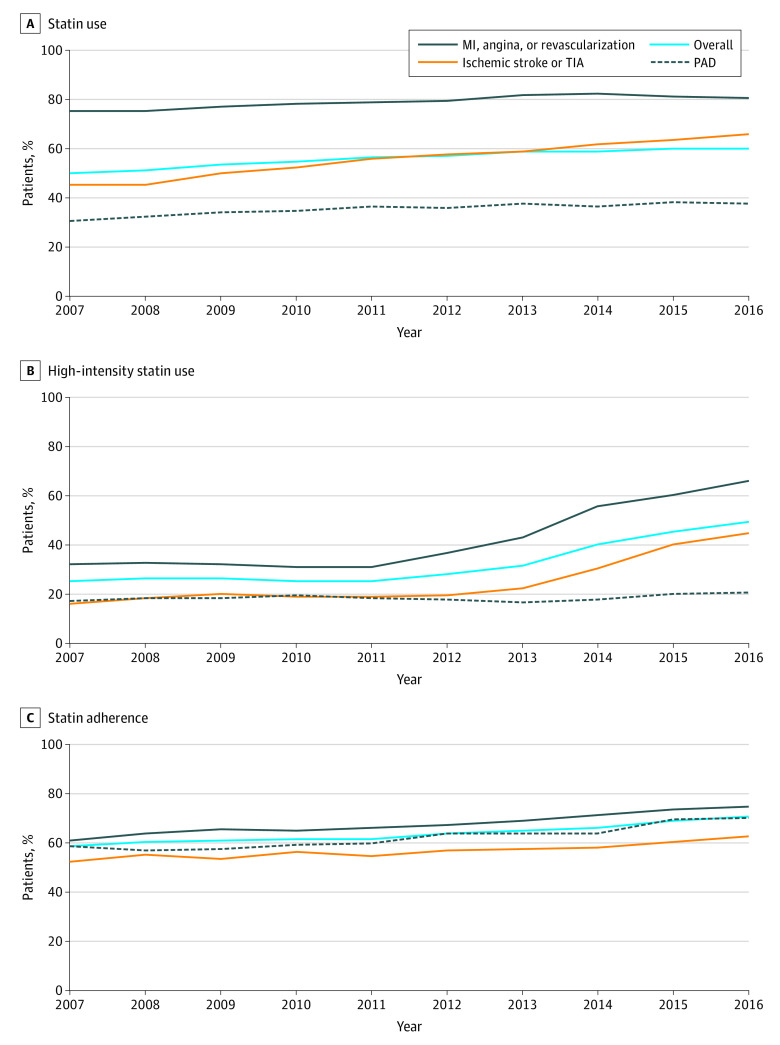
Trends of Statin Use and Adherence, Stratified by Atherosclerotic Cardiovascular Disease Type A, The proportion of patients who used statin within 30 days of discharge. B, The proportion of patients who used a high-intensity statin among statin users. C, The proportion of patients who adhered to statins at the end of 1 year among statin users who had at least 1 year of follow-up. Adherence was defined as proportion of days covered greater than or equal to 80%. MI indicates myocardial infarction; PAD, peripheral artery disease; TIA, transient ischemic attack.

Statin use within 30 days of discharge increased from 50.3% in 2007 to 59.9% in 2016 (*P* < .001) (Figure 1; eTable 4 in the Supplement). Statin use within 90 days of discharge increased from 52.6% in 2007 to 60.3% in 2016 (*P* < .001) (eTable 5 in the Supplement). Statin use was higher in 2016 in patients with CHD (80.9%) than patients with ischemic stroke or TIA (65.8%), or PAD (37.5%). In the multivariable regression analysis, patients aged 65 to 74 years were more likely to receive statins (adjusted odds ratio [aOR], 1.26; 95% CI, 1.24-1.28; *P* < .001) than patients aged less than 65 years, whereas patients aged 75 years or older were less likely to receive statins (aOR, 0.95; 95% CI, 0.94, 0.97; *P* < .001). Women were less likely to receive statins than men (aOR, 0.81; 95% CI, 0.80-0.82; *P* < .001). Black and Hispanic patients were less likely to receive statins than White patients (for Black patients: aOR, 0.90; 95% CI, 0.88-0.93; *P* < .001; for Hispanic patients: aOR, 0.90; 95% CI, 0.88-0.92; *P* < .001) (Table 2).

**Table 2.** Patient Characteristics Associated With Statin Use Within 30 Days of Discharge

Characteristic	% Used statin	Adjusted OR (95% CI)^a^	*P* value
Age, y			
<65	55.3	1 [Reference]	NA
65-74	60.9	1.26 (1.24-1.28)	<.001
≥75	52.1	0.95 (0.94-0.97)	<.001
Sex			
Male	61.7	1 [Reference]	NA
Female	49.7	0.81 (0.80-0.82)	<.001
Race			
Asian	54.8	1.07 (1.03-1.12)	.002
Black	52.5	0.90 (0.88-0.93)	<.001
Hispanic	50.5	0.90 (0.88-0.92)	<.001
White	57.7	1 [Reference]	NA
Other/unknown	56.9	0.98 (0.94-1.03)	.39
ASCVD type			
MI, angina, or revascularization	79.2	1 [Reference]	NA
Ischemic stroke or TIA	55.5	0.34 (0.33-0.35)	<.001
PAD	35.4	0.14 (0.14-0.15)	<.001
Hypertension			
No	42.0	1 [Reference]	NA
Yes	59.2	1.77 (1.74-1.81)	<.001
Diabetes			
No	52.9	1 [Reference]	NA
Yes	62.1	1.47 (1.45-1.50)	<.001
Stage 3-5 CKD			
No	55.7	1 [Reference]	NA
Yes	59.9	1.04 (1.01-1.06)	.006
Heart failure			
No	55.0	1 [Reference]	NA
Yes	62.3	0.84 (0.82-0.86)	<.001
Index year			
2007	50.3	1 [Reference]	NA
2008	51.2	1.04 (1.01-1.08)	.02
2009	53.6	1.17 (1.13-1.21)	<.001
2010	55.0	1.25 (1.21-1.29)	<.001
2011	56.7	1.36 (1.31-1.41)	<.001
2012	57.0	1.38 (1.34-1.43)	<.001
2013	58.7	1.51 (1.46-1.56)	<.001
2014	59.1	1.54 (1.49-1.60)	<.001
2015	59.8	1.59 (1.54-1.64)	<.001
2016	59.9	1.60 (1.55-1.66)	<.001

Abbreviations: ASCVD, atherosclerotic cardiovascular disease; CKD, chronic kidney disease; NA, not applicable; OR, odds ratio; PAD, peripheral artery disease; TIA, transient ischemic attack.

^a^ Adjusted odds ratios were obtained from a multivariable logistic regression with statin use as the outcome variable and all these baseline characteristics as independent variables.

The use of a high-intensity statin increased from 25.0% in 2007 to 49.2% in 2016 (*P* < .001) (Figure 1; eTable 6 in the Supplement), which increased substantially after 2013 when the ACC/AHA guidelines were released. However, the increase in patients with PAD occurred more slowly than the increase in patients in other groups. In 2016, among patients who used statins, 66.0% of patients with CHD used a high-intensity statin vs 45.1% of patients with ischemic stroke or TIA and 20.4% of patients with PAD. In the multivariable regression analysis, patients aged 65 to 74 years and aged 75 years or older were less likely to receive high-intensity statins than patients aged less than 65 years (age 65-74 years: OR, 0.77; 95% CI, 0.75-0.79; *P* < .001; age ≥75 years: OR, 0.56; 95% CI, 0.54-0.58; *P* < .001). Women were less likely to receive high-intensity statins than men (OR, 0.79; 95% CI, 0.77-0.81: *P* < .001) (eTable 7 in the Supplement).

The adherence to statin therapy at the end of 1 year increased from 58.7% of patients with PDC of at least 80% in 2007 to 70.5% in 2016 (*P* < .001) (Figure 1; eTable 8 in the Supplement). In the multivariable regression analysis, patients aged 65 to 74 years and aged 75 years or older were more likely to adhere to medications than patients aged less than 65 years (age 65-74 years: OR, 1.38; 95% CI, 1.34-1.41; *P* < .001; age ≥75 years: OR, 1.47; 95% CI, 1.43-1.52;, *P* < .001). Women were less likely to adhere to medications than men (OR, 0.86; 95% CI, 0.84-0.88; *P* < .001). Black, Hispanic, and Asian individuals were less likely to adhere to medications than White individuals (for Black individuals: OR, 0.61; 95% CI, 0.59-0.64; *P* < .001; for Hispanic individuals: OR, 0.60; 95% CI, 0.58-0.63; *P* < .001; for Asian individuals: OR, 0.86; 95% CI, 0.81-0.92; *P* < .001) (eTable 9 in the Supplement).

### Statin Costs

**Figure 2.**
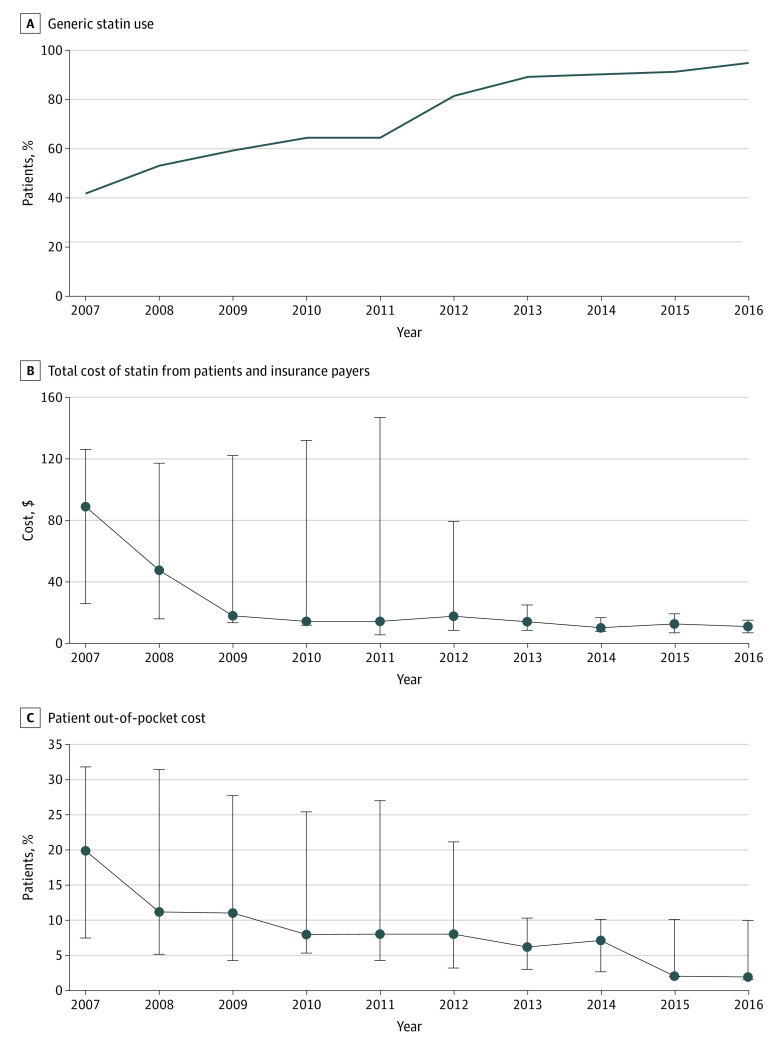
Trends of Use of Generic Statin and Statin Cost A, The proportion of statin users who used a generic statin. B, The total cost of statin. C, The out-of-pocket cost of statin. The costs were adjusted to reflect cost for a 30-day supply in 2016 US dollars and were presented as median. The error bars indicate the interquartile range.

Among individuals who used statin, the proportion of patients using a generic statin increased from 42.0% in 2007 to 94.9% in 2016 (*P* < .001) (Figure 2). The median total cost per month decreased from $88.9 (IQR, $25.6-$126.0) in 2007 to $10.8 (IQR, $6.9-$15.0) in 2016 (*P* < .001), and the median out-of-pocket cost decreased from $20.0 (IQR, $7.6-$31.9) in 2007 to $2.0 (IQR, $1.6-$10.0) in 2016 (*P* < .001 for all trends) (eTable 10 in the Supplement).

### Clinical Outcomes

**Figure 3.**
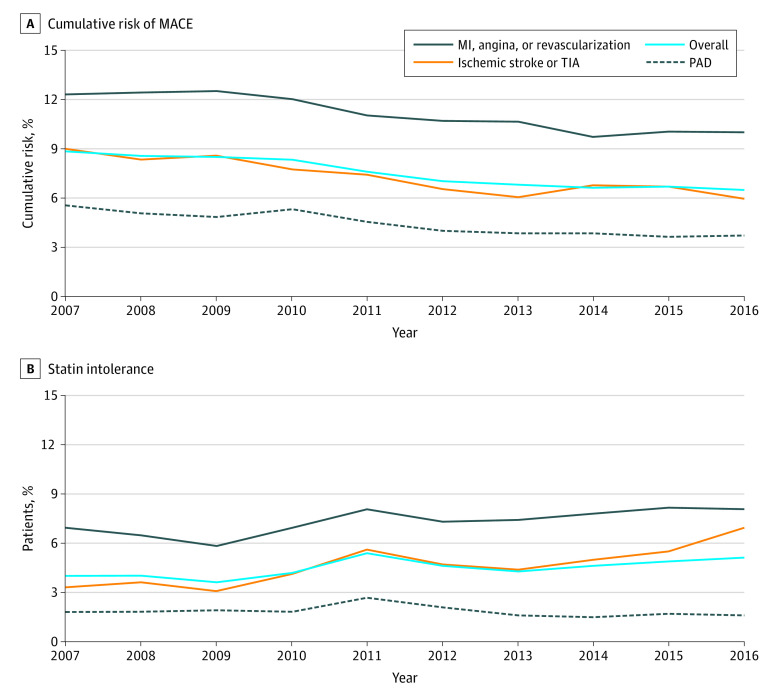
Trends of Outcomes, Stratified by Atherosclerotic Cardiovascular Disease Type A, Cumulative risk of major adverse cardiac events (MACE) at 1 year, including myocardial infarction, ischemic stroke, revascularization, and all-cause mortality. B, The proportion of patients who developed statin intolerance within 1 year among patients who had at least 1 year of follow-up. MI indicates myocardial infarction; PAD, peripheral artery disease; TIA, transient ischemic attack.

The 1-year risk of MACE decreased from 8.9% in 2007 to 6.5% in 2016 (*P* < .001) (Figure 3; eTable 11 in the Supplement). Statin use within 30 days of discharge was associated with a lower risk of MACE in the overall cohort (hazard ratio [HR], 0.90; 95% CI, 0.86-0.94; *P* < .001) and in each of the 3 groups (for patients with CHD: HR, 0.87; 95% CI, 0.84-0.91; *P* < .001; for patients with ischemic stroke or TIA: HR; 0.93; 95% CI, 0.88- 0.98; *P* = .01; for patients with PAD: HR, 0.86; 95% CI, 0.82-0.90; *P* < .001). There was no significant interaction between statin treatment and ASCVD type (eTable 12 in the Supplement). In patients with PAD, the risk of lower extremity vascular complications was consistent from 4.9% in 2007 to 4.8% in 2016 (eFigure 2 in the Supplement).

The proportion of patients who had statin intolerance increased from 4.0% in 2007 to 5.1% in 2016 (Figure 3; eTable 13 in the Supplement). The increase was greater in patients with ischemic stroke or TIA (3.3% in 2007 to 6.9% in 2016), whereas statin intolerance did not increase in patients with PAD (1.8% in 2007 and 1.6% in 2016). The trends of use, adherence, and clinical outcomes by age and sex in the various ASCVD types were generally consistent with the overall cohort (eTables 14-21 and eFigures 3-6 in the Supplement). For example, in patients with CHD and aged less than 65 years who initiated a statin, the use of high-intensity statin increased from 34.8% in 2007 to 73.5% in 2016 (*P* < .001). In older adults, the use of high-intensity statin was lower: in 2016, 59% of patients with CHD and aged 65 years or older who initiated a statin used a high-intensity statin.

## Discussion

This study assessed trends in the use, adherence, cost, and outcomes of statin therapy for secondary prevention of ASCVD from 2007 to 2016. There appears to be a modest increase in statin use and adherence, whereas the use of high-intensity statins approximately doubled over this time frame. The 1-year MACE risk decreased from 8.9% to 6.5%, translating to 525 600 fewer MACE events per year assuming there are approximately 21.9 million US residents with ASCVD.^1^ Although observational data cannot establish a causal relationship, these trends suggest an improvement in clinical practice over time.

The results in patients with CHD were consistent with prior studies. For example, in a study of Medicare patients with acute MI,^11^ 83% of patients initiated statin within 30 days of the event (80.9% of patients with CHD in 2016 in this study). In another study of patients post-MI, ^12^ 72% of the patients who used statin were less than age 65 years and 58% of the patients who used statin aged 65 years or older received high-intensity statins (73.5% and 59% in 2016 in this study).

However, these previous studies are largely limited to a specific population, eg, patient with CHD or within a specific region, health system, or insurance plan.^12,25,26,27,28,29,30^ To our knowledge, this is the first study to assess the contemporary trends in a large national cohort of patients both less than or more than age 65 years with various ASCVD types receiving care in diverse routine practice settings. The findings highlight major treatment gaps that warrant future study.

First, the use of statins, high-intensity statins, and adherence to statins were all lower in patients with ischemic stroke, TIA, and PAD compared with those with a history of CHD. Statin use was particularly low in patients with PAD: only 1 in 3 patients with PAD received a statin and 1 in 15 received a high-intensity statin. The use in this cohort was lower than what was reported in previous literature perhaps because this study included a patient’s first ASCVD event, and none of these patients with PAD had a history of CHD or stroke, whereas other PAD cohorts contained 50% to 75% of patients with other types of ASCVD.^31,32^ In a previous study of asymptomatic PAD patients without cardiovascular disease, only 1 in 4 used a statin.^33^

This gap in treatment and adherence might reflect patient and physician perception that patients without CHD have a lower risk of subsequent MACE, and thus need less aggressive treatment. Most randomized clinical trials demonstrating the efficacy of statins were conducted in patients with CHD, which may help explain the less aggressive treatment. However, as shown in this study and a previous study,^32^ the lower risk of MACE associated with statin use was consistent across different ASCVD subgroups.

Second, women and Black, Hispanic, and Asian individuals are less likely to receive and adhere to statin therapy. After adjusting for clinical characteristics, women are approximately 20% less likely to receive statins or high-intensity statins than men and 14% less likely to adhere to medications; Black and Hispanic patients were 10% less likely to receive statins and 40% less likely to adhere to medications.

Another key finding is that the cost of statin therapy decreased substantially over time, which may be associated with the availability and the increasing use of generic statins. In 2016, the median out-of-pocket cost per 30-day supply was only $2. However, despite the decrease in costs of treatment, the adherence to statin therapy only modestly increased and nearly 30% of patients did not adhere to statins within 1 year. This finding suggests that many patients may have barriers to adherence unrelated to costs, and innovative strategies will need to be explored to help improve adherence in such patients.^34^

Statin intolerance increases from 4% to 2007 to 5% in 2016. The increase was particularly evident in patients with ischemic stroke or TIA, among whom there was a substantial increase of high-intensity statin use, whereas there was no increase in statin intolerance in patients with PAD. For most patients, the benefit of secondary prevention outweighs the risk of statin intolerance. However, statin intolerance can affect a person’s perceived quality of life and may need to be considered during shared decision-making.^35,36^

### Limitations

The study has several limitations. First, administrative claims data are subject to misclassification. However, the algorithms used in this study have been commonly used in previous studies and demonstrated good performance in validation studies.^19,37,38,39,40^ Second, the study included only privately insured and Medicare Advantage patients. However, the insurance coverage rate is high in older US residents with ASCVD. More than 90% of US residents aged 50 to 64 years have health insurance and more than 75% had private health insurance.^41^ One in 3 Medicare patients is enrolled in Medicare Advantage.^42^ Although traditionally Medicare Advantage attracted healthier people, after the risk adjustment system was phased in from 2004 to 2007, the favorable risk selection has been largely reduced.^43^ The distribution of age, sex, and race/ethnicity in OptumLabs enrollees was almost the same as a nationally representative cohort from the Medical Expenditure Panel Survey.^1^

## Conclusions

The results of this cohort study found that from 2007 to 2016, modest improvements in the use, adherence, and cardiovascular outcomes for statin therapy in patients with ASCVD have occurred, but a substantial and persistent treatment gap exists between patients with and without CHD, between men and women. Many patients with PAD, ischemic stroke, and TIA and women remain undertreated.

## Notes

Supplement. **eTable 1.** Comparison of OptumLabs Data and a US Nationally Representative Cohort **eTable 2.** Patient Characteristics Across Different Years Without Weight **eTable 3.** Patient Characteristics Across Different Years With Weight **eTable 4.** Percentages of Patients Using Statins Within 30 Days of Discharge With and Without Weights **eTable 5.** Percentages of Patients Using Statins Within 90 Days of Discharge **eTable 6.** Percentages of Patients Using High-Intensity Statins Among Patients Who Used Statins Within 30 Days of Discharge **eTable 7.** Patient Characteristics Associated With High-Intensity Statin Use **eTable 8.** Percentages of Patients With PDC ≥80% Among Patients Who Used Statins Within 30 Days of Discharge **eTable 9.** Patient Characteristics Associated With Adherence **eTable 10.** Percentages of Patients Using a Generic Statin and Cost per 30-Day Supply **eTable 11.** Cumulative Risk of Major Adverse Cardiac Event at One Year (%) **eTable 12.** Association Between Statin Use Within 30 Days of Discharge and MACE **eTable 13.** Statin Intolerance Within One Year (%) **eTable 14.** Trends of Statin Use Within 30 Days of Discharge (%), Stratified by ASCVD Type and Age **eTable 15.** Trends of High-Intensity Statin Use Within 30 Days of Discharge (%), Stratified by ASCVD Type and Age **eTable 16.** Trends of One-Year Statin Adherence (%), Stratified by ASCVD Type and Age **eTable 17.** Trends of One-Year Risk of MACE (%), Stratified by ASCVD Type and Age **eTable 18.** Trends of Statin Use Within 30 Days of Discharge (%), Stratified by ASCVD Type and Sex **eTable 19.** Trends of High-Intensity Statin Use Within 30 Days of Discharge (%), Stratified by ASCVD Type and Sex **eTable 20.** Trends of One-Year Statin Adherence (%), Stratified by ASCVD Type and Sex **eTable 21.** Trends of One-Year risk of MACE (%), Stratified by ASCVD Type and Sex **eFigure 1.** Patient Selection Flow Diagram **eFigure 2.** One-Year Cumulative Risk of Lower Extremity Vascular Complications Among Patients With PAD **eFigure 3.** Trends of Statin Use, Stratified by ASCVD Type and Age **eFigure 4.** Trends of Statin Use, Stratified by ASCVD Type and Sex **eFigure 5.** Trends in One-Year Risk of MACE (%), Stratified by ASCVD Type and Age **eFigure 6.** Trends in One-Year Risk of MACE (%), Stratified by ASCVD Type and Sex **eAppendix.** Statistical Analysis Plan **eReferences**

## References

[1] Salami JA, Warraich H, Valero-Elizondo J, National trends in statin use and expenditures in the US adult population from 2002 to 2013: insights from the Medical Expenditure Panel Survey. JAMA Cardiol. 2017;2(1):56-65. doi:10.1001/jamacardio.2016.4700 27842171

[2] Benjamin EJ, Muntner P, Alonso A, ; American Heart Association Council on Epidemiology and Prevention Statistics Committee and Stroke Statistics Subcommittee Heart disease and stroke statistics-2019 update: a Report from the American Heart Association. Circulation. 2019;139(10):e56-e528. doi:10.1161/CIR.0000000000000659 30700139

[3] Baigent C, Blackwell L, Emberson J, ; Cholesterol Treatment Trialists’ (CTT) Collaboration Efficacy and safety of more intensive lowering of LDL cholesterol: a meta-analysis of data from 170,000 participants in 26 randomised trials. Lancet. 2010;376(9753):1670-1681. doi:10.1016/S0140-6736(10)61350-5 21067804 PMC2988224

[4] Shah ND, Dunlay SM, Ting HH, Long-term medication adherence after myocardial infarction: experience of a community. Am J Med. 2009;122(10):961.e7-961.e13. doi:10.1016/j.amjmed.2008.12.021 19560749 PMC3771524

[5] Chowdhury R, Khan H, Heydon E, Adherence to cardiovascular therapy: a meta-analysis of prevalence and clinical consequences. Eur Heart J. 2013;34(38):2940-2948. doi:10.1093/eurheartj/eht295 23907142

[6] Rasmussen JN, Chong A, Alter DA Relationship between adherence to evidence-based pharmacotherapy and long-term mortality after acute myocardial infarction. JAMA. 2007;297(2):177-186. doi:10.1001/jama.297.2.177 17213401

[7] De Vera MA, Bhole V, Burns LC, Lacaille D Impact of statin adherence on cardiovascular disease and mortality outcomes: a systematic review. Br J Clin Pharmacol. 2014;78(4):684-698. doi:10.1111/bcp.12339 25364801 PMC4239963

[8] Rodriguez F, Maron DJ, Knowles JW, Virani SS, Lin S, Heidenreich PA Association of statin adherence with mortality in patients with atherosclerotic cardiovascular disease. JAMA Cardiol. 2019;4(3):206-213. doi:10.1001/jamacardio.2018.4936 30758506 PMC6439552

[9] van Driel ML, Morledge MD, Ulep R, Shaffer JP, Davies P, Deichmann R Interventions to improve adherence to lipid-lowering medication. Cochrane Database Syst Rev. 2016;12:CD004371. doi:10.1002/14651858.CD004371.pub4 28000212 PMC6464006

[10] Stone NJ, Robinson JG, Lichtenstein AH, ; American College of Cardiology/American Heart Association Task Force on Practice Guidelines 2013 ACC/AHA guideline on the treatment of blood cholesterol to reduce atherosclerotic cardiovascular risk in adults: a report of the American College of Cardiology/American Heart Association Task Force on Practice Guidelines. J Am Coll Cardiol. 2014;63(25 Pt B)(25, pt B):2889-2934. doi:10.1016/j.jacc.2013.11.002 24239923

[11] Korhonen MJ, Robinson JG, Annis IE, Adherence tradeoff to multiple preventive therapies and all-cause mortality after acute myocardial infarction. J Am Coll Cardiol. 2017;70(13):1543-1554. doi:10.1016/j.jacc.2017.07.783 28935030 PMC5890809

[12] Rosenson RS, Farkouh ME, Mefford M, Trends in use of high-intensity statin therapy after myocardial infarction, 2011 to 2014. J Am Coll Cardiol. 2017;69(22):2696-2706. doi:10.1016/j.jacc.2017.03.585 28571633

[13] Wallace PJ, Shah ND, Dennen T, Bleicher PA, Crown WH Optum Labs: building a novel node in the learning health care system. Health Aff (Millwood). 2014;33(7):1187-1194. doi:10.1377/hlthaff.2014.0038 25006145

[14] Optum. Optum Research Data Assets, 2015 Accessed October 22, 2020. https://www.optum.com/content/dam/optum/resources/productSheets/5302_Data_Assets_Chart_Sheet_ISPOR.pdf

[15] Yao X, Gersh BJ, Holmes DR Jr, Association of surgical left atrial appendage occlusion with subsequent stroke and mortality among patients undergoing cardiac surgery. JAMA. 2018;319(20):2116-2126. doi:10.1001/jama.2018.6024 29800182 PMC6351069

[16] Maraka S, Mwangi R, McCoy RG, Thyroid hormone treatment among pregnant women with subclinical hypothyroidism: US national assessment. BMJ. 2017;356:i6865. Published online January 25, 2017. doi:10.1136/bmj.i6865 28122781 PMC5266622

[17] McCoy RG, Van Houten HK, Ross JS, Montori VM, Shah ND HbA1c overtesting and overtreatment among US adults with controlled type 2 diabetes, 2001-13: observational population based study. BMJ. 2015;351:h6138. Published online December 8, 2015. doi:10.1136/bmj.h6138 26646052 PMC4673101

[18] Kernan WN, Ovbiagele B, Black HR, ; American Heart Association Stroke Council, Council on Cardiovascular and Stroke Nursing, Council on Clinical Cardiology, and Council on Peripheral Vascular Disease Guidelines for the prevention of stroke in patients with stroke and transient ischemic attack: a guideline for healthcare professionals from the American Heart Association/American Stroke Association. Stroke. 2014;45(7):2160-2236. doi:10.1161/STR.0000000000000024 24788967

[19] Fan J, Arruda-Olson AM, Leibson CL, Billing code algorithms to identify cases of peripheral artery disease from administrative data. J Am Med Inform Assoc. 2013;20(e2):e349-e354. doi:10.1136/amiajnl-2013-001827 24166724 PMC3861931

[20] Hershman DL, Tsui J, Wright JD, Coromilas EJ, Tsai WY, Neugut AI Household net worth, racial disparities, and hormonal therapy adherence among women with early-stage breast cancer. J Clin Oncol. 2015;33(9):1053-1059. doi:10.1200/JCO.2014.58.3062 25691670 PMC4356713

[21] Dunn A, Grosse SD, Zuvekas SH Adjusting health expenditures for inflation: a review of measures for health services research in the United States. Health Serv Res. 2018;53(1):175-196. doi:10.1111/1475-6773.12612 27873305 PMC5785315

[22] Agency for Healthcare Research and Quality Medical Expenditure Panel Survey: Using appropriate price indices for analyses of health care expenditures or income across multiple years. Accessed October 22, 2020. https://meps.ahrq.gov/about_meps/Price_Index.shtml

[23] Serban MC, Colantonio LD, Manthripragada AD, Statin Intolerance and risk of coronary heart events and all-cause mortality following myocardial infarction. J Am Coll Cardiol. 2017;69(11):1386-1395. doi:10.1016/j.jacc.2016.12.036 28302290

[24] Colantonio LD, Kent ST, Huang L, Algorithms to identify statin intolerance in Medicare administrative claim data. Cardiovasc Drugs Ther. 2016;30(5):525-533. doi:10.1007/s10557-016-6680-3 27389413

[25] Rosenson RS, Kent ST, Brown TM, Underutilization of high-intensity statin therapy after hospitalization for coronary heart disease. J Am Coll Cardiol. 2015;65(3):270-277. doi:10.1016/j.jacc.2014.09.088 25614424

[26] Bin Abdulhak AA, Vaughan-Sarrzin M, Kaboli P, Temporal trends of high-intensity statin therapy among veterans treated with percutaneous coronary intervention. J Am Heart Assoc. 2018;7(5):e007370. doi:10.1161/JAHA.117.007370 29503265 PMC5866316

[27] Peters SAE, Colantonio LD, Zhao H, Sex differences in high-intensity statin use following myocardial infarction in the United States. J Am Coll Cardiol. 2018;71(16):1729-1737. doi:10.1016/j.jacc.2018.02.032 29673463

[28] Bittner V, Colantonio LD, Dai Y, Association of region and hospital and patient characteristics with use of high-intensity statins after myocardial infarction among Medicare beneficiaries. JAMA Cardiol. 2019;4(9):865-872. doi:10.1001/jamacardio.2019.2481 31339519 PMC6659160

[29] Albright KC, Howard VJ, Howard G, Age and sex disparities in discharge statin prescribing in the Stroke Belt: evidence from the Reasons for Geographic and Racial Differences in Stroke Study. J Am Heart Assoc. 2017;6(8):e005523. doi:10.1161/JAHA.117.005523 28768644 PMC5586419

[30] Xian Y, Navar AM, Li S, Intensity of lipid lowering with statin therapy in patients with cerebrovascular disease versus coronary artery disease: insights from the PALM Registry. J Am Heart Assoc. 2019;8(19):e013229. doi:10.1161/JAHA.119.013229 31554462 PMC6806030

[31] Kumbhani DJ, Steg PG, Cannon CP, ; REACH Registry Investigators Statin therapy and long-term adverse limb outcomes in patients with peripheral artery disease: insights from the REACH registry. Eur Heart J. 2014;35(41):2864-2872. doi:10.1093/eurheartj/ehu080 24585266 PMC4216432

[32] Arya S, Khakharia A, Binney ZO, Association of statin dose with amputation and survival in patients with peripheral artery disease. Circulation. 2018;137(14):1435-1446. doi:10.1161/CIRCULATIONAHA.117.032361 29330214 PMC5882502

[33] Ramos R, García-Gil M, Comas-Cufí M, Statins for prevention of cardiovascular events in a low-risk population with low ankle brachial index. J Am Coll Cardiol. 2016;67(6):630-640. doi:10.1016/j.jacc.2015.11.052 26868687

[34] Banach M, Stulc T, Dent R, Toth PP Statin non-adherence and residual cardiovascular risk: There is need for substantial improvement. Int J Cardiol. 2016;225:184-196. doi:10.1016/j.ijcard.2016.09.075 27728862

[35] Rosenson RS, Baker S, Banach M, Optimizing cholesterol treatment in patients with muscle complaints. J Am Coll Cardiol. 2017;70(10):1290-1301. doi:10.1016/j.jacc.2017.07.752 28859793

[36] Banach M, Rizzo M, Toth PP, Statin intolerance - an attempt at a unified definition. Position paper from an International Lipid Expert Panel. Arch Med Sci. 2015;11(1):1-23. doi:10.5114/aoms.2015.49807 25861286 PMC4379380

[37] Kumamaru H, Judd SE, Curtis JR, Validity of claims-based stroke algorithms in contemporary Medicare data: reasons for geographic and racial differences in stroke (REGARDS) study linked with Medicare claims. Circ Cardiovasc Qual Outcomes. 2014;7(4):611-619. doi:10.1161/CIRCOUTCOMES.113.000743 24963021 PMC4109622

[38] Tirschwell DL, Longstreth WT Jr Validating administrative data in stroke research. Stroke. 2002;33(10):2465-2470. doi:10.1161/01.STR.0000032240.28636.BD 12364739

[39] Kokotailo RA, Hill MD Coding of stroke and stroke risk factors using international classification of diseases, revisions 9 and 10. Stroke. 2005;36(8):1776-1781. doi:10.1161/01.STR.0000174293.17959.a1 16020772

[40] Jensen PN, Johnson K, Floyd J, Heckbert SR, Carnahan R, Dublin S Identifying atrial fibrillation from electronic medical data: a systematic review. Pharmacoepidemiol Drug Saf. 2012;21:141-147. doi:10.1002/pds.2317 22262600 PMC3674852

[41] Barnett JC, Vornovitsky MS Health Insurance Coverage in the United States: 2015. US Government Printing Office; 2016.

[42] Jacobson G, Casillas G, Damico A, Neuman T, Gold M Medicare Advantage 2016 spotlight: enrollment market update. Published May 11, 2016 Accessed October 22, 2020. https://www.kff.org/report-section/medicare-advantage-2016-spotlight-enrollment-market-update-appendices/

[43] McWilliams JM, Hsu J, Newhouse JP New risk-adjustment system was associated with reduced favorable selection in Medicare Advantage. Health Aff (Millwood). 2012;31(12):2630-2640. doi:10.1377/hlthaff.2011.1344 23213147 PMC3538078

