# Supplementary material for: Assessment of Trends in Statin Therapy for Secondary Prevention of Atherosclerotic Cardiovascular Disease in US Adults From 2007 to 2016
Source: JAMA Netw Open. 2020 Nov 20;3(11):e2025505. doi: 10.1001/jamanetworkopen.2020.25505 (PMC7679951; doi:10.1001/jamanetworkopen.2020.25505)
Supplement: Supplement. — eTable 1. Comparison of OptumLabs Data and a US Nationally Representative Cohort eTable 2. Patient Characteristics Across Different Years Without Weight eTable 3. Patient Characteristics Across Different Years With Weight eTable 4. Percentages of Patients Using Statins Within 30 Days of Discharge With and Without Weights eTable 5. Percentages of Patients Using Statins Within 90 Days of Discharge eTable 6. Percentages of Patients Using High-Intensity Statins Among Patients Who Used Statins Within 30 Days of Discharge eTable 7. Patient Characteristics Associated With High-Intensity Statin Use eTable 8. Percentages of Patients With PDC ≥80% Among Patients Who Used Statins Within 30 Days of Discharge eTable 9. Patient Characteristics Associated With Adherence eTable 10. Percentages of Patients Using a Generic Statin and Cost per 30-Day Supply eTable 11. Cumulative Risk of Major Adverse Cardiac Event at One Year (%) eTable 12. Association Between Statin Use Within 30 Days of Discharge and MACE eTable 13. Statin Intolerance Within One Year (%) eTable 14. Trends of Statin Use Within 30 Days of Discharge (%), Stratified by ASCVD Type and Age eTable 15. Trends of High-Intensity Statin Use Within 30 Days of Discharge (%), Stratified by ASCVD Type and Age eTable 16. Trends of One-Year Statin Adherence (%), Stratified by ASCVD Type and Age eTable 17. Trends of One-Year Risk of MACE (%), Stratified by ASCVD Type and Age eTable 18. Trends of Statin Use Within 30 Days of Discharge (%), Stratified by ASCVD Type and Sex eTable 19. Trends of High-Intensity Statin Use Within 30 Days of Discharge (%), Stratified by ASCVD Type and Sex eTable 20. Trends of One-Year Statin Adherence (%), Stratified by ASCVD Type and Sex eTable 21. Trends of One-Year risk of MACE (%), Stratified by ASCVD Type and Sex eFigure 1. Patient Selection Flow Diagram eFigure 2. One-Year Cumulative Risk of Lower Extremity Vascular Complications Among Patients With PAD eFigure 3. Trends of Statin Use, Stratified by A [file jamanetwopen-e2025505-s001.pdf]

Supplemental Online Content

Yao X, Shah ND, Gersh BJ, Lopez-Jimenez F, Noseworthy PA. Assessment of trends in statin therapy for secondary prevention of atherosclerotic cardiovascular disease in US adults from 2007 to 2016. *JAMA Netw Open*. 2020;3(11):e2025505.
doi:10.1001/jamanetworkopen.2020.25505

eTable 1. Comparison of OptumLabs Data and a US Nationally Representative Cohort

eTable 2. Patient Characteristics Across Different Years Without Weight

eTable 3. Patient Characteristics Across Different Years With Weight

eTable 4. Percentages of Patients Using Statins Within 30 Days of Discharge With and Without Weights

eTable 5. Percentages of Patients Using Statins Within 90 Days of Discharge

eTable 6. Percentages of Patients Using High-Intensity Statins Among Patients Who Used Statins Within 30 Days of Discharge

eTable 7. Patient Characteristics Associated With High-Intensity Statin Use

eTable 8. Percentages of Patients With PDC ⩾80% Among Patients Who Used Statins Within 30 Days of Discharge

eTable 9. Patient Characteristics Associated With Adherence

eTable 10. Percentages of Patients Using a Generic Statin and Cost per 30-Day Supply

eTable 11. Cumulative Risk of Major Adverse Cardiac Event at One Year (%)

eTable 12. Association Between Statin Use Within 30 Days of Discharge and MACE

eTable 13. Statin Intolerance Within One Year (%)

eTable 14. Trends of Statin Use Within 30 Days of Discharge (%), Stratified by ASCVD Type and Age

eTable 15. Trends of High-Intensity Statin Use Within 30 Days of Discharge (%), Stratified by ASCVD Type and Age

eTable 16. Trends of One-Year Statin Adherence (%), Stratified by ASCVD Type and Age

eTable 17. Trends of One-Year Risk of MACE (%), Stratified by ASCVD Type and Age

© 2020 Yao X et al. *JAMA Network Open*.

eTable 18. Trends of Statin Use Within 30 Days of Discharge (%), Stratified by ASCVD Type and Sex

eTable 19. Trends of High-Intensity Statin Use Within 30 Days of Discharge (%), Stratified by ASCVD Type and Sex

eTable 20. Trends of One-Year Statin Adherence (%), Stratified by ASCVD Type and Sex

eTable 21. Trends of One-Year risk of MACE (%), Stratified by ASCVD Type and Sex

eFigure 1. Patient Selection Flow Diagram

eFigure 2. One-Year Cumulative Risk of Lower Extremity Vascular Complications Among Patients With PAD

eFigure 3. Trends of Statin Use, Stratified by ASCVD Type and Age

eFigure 4. Trends of Statin Use, Stratified by ASCVD Type and Sex

eFigure 5. Trends in One-Year Risk of MACE (%), Stratified by ASCVD Type and Age

eFigure 6. Trends in One-Year Risk of MACE (%), Stratified by ASCVD Type and Sex

eAppendix. Statistical Analysis Plan

eReferences

This supplemental material has been provided by the authors to give readers additional information about their work.

© 2020 Yao X et al. *JAMA Network Open*.

eTable 1. Comparison of OptumLabs Data and a US Nationally Representative Cohort

	OptumLabs Cohort	Nationally Representative Cohort
**Age, mean, y**	58.3	58.5
**Age category, y**		
<65	69.4	69.9
65-74	18.4	17.4
≥75	12.1	12.7
**Sex**		
Male	47.6	47.8
Female	52.4	52.2
**Race/ethnicity**		
Asian	3.9	4.9
Black	10.6	10.8
Hispanic	8.4	11.4
White	74.5	71.1
Other/unknown	2.7	1.8

*OptumLabs includes patients of all ages and races from all 50 states. To demonstrate the similarities between the OptumLabs cohort and a nationally representative cohort, we used 4,991,144 people aged ≥40 years from the OptumLabs cohort in 2013 and compared them with a nationally representative cohort aged ≥40 years in a previous study using the Medical Expenditure Panel Survey (MEPS) 2012-2013 data (Salami et al. JAMA Cardiol. 2017;2(1):56-65)

© 2020 Yao X et al. *JAMA Network Open*.

eTable 2. Patient characteristics across different years without weight

Characteristics	2007 (N=28501)	2008 (N=28431)	2009 (N=28527)	2010 (N=28507)	2011 (N=28478)	2012 (N=28491)	2013 (N=28504)	2014 (N=28502)	2015 (N=28506)	2016 (N=28506)
Age - yr										
Median	58	58	59	61	62	63	66	66	67	67
Interquartile range	51-66	50-66	51-67	52-70	53-71	54-73	56-74	56-74	57-75	57-75
Female sex – no. (%)	9282(42.4)	10037(42.2)	11186(44.1)	12181(44.9)	12941(45.1)	13984(45.8)	15549(46.3)	13243(45.0)	14580(46.5)	15439(46.4)
Race										
Asian	440(2.0)	496(2.1)	587(2.3)	638(2.3)	716(2.5)	782(2.6)	1364(4.1)	1148(3.9)	1151(3.7)	1145(3.4)
Black	1900(8.7)	2359(9.9)	2930(11.5)	3354(12.4)	3640(12.7)	4265(14.0)	4579(13.6)	3693(12.6)	3734(11.9)	4255(12.8)
Hispanic	1651(7.5)	1755(7.4)	2069(8.2)	2100(7.7)	2167(7.6)	2376(7.8)	3110(9.3)	2810(9.6)	3373(10.8)	3503(10.5)
White	16748(76.5)	18216(76.7)	18944(74.7)	20297(74.8)	21308(74.3)	22250(72.9)	23564(70.2)	20882(71.0)	22217(70.8)	23355(70.2)
Other/unknown	1165(5.3)	932(3.9)	843(3.3)	762(2.8)	834(2.9)	836(2.7)	939(2.8)	873(3.0)	897(2.9)	1002(3.0)
Index event – no. (%)										
MI, Angina or Revascularization	9251(42.2)	9733(41.0)	9555(37.7)	9905(36.5)	10387(36.2)	10910(35.8)	11265(33.6)	10252(34.9)	11059(35.3)	12183(36.6)
Ischemic stroke or TIA	4438(20.3)	4825(20.3)	5194(20.5)	5480(20.2)	6012(21.0)	6591(21.6)	7181(21.4)	6418(21.8)	7057(22.5)	7670(23.1)
PAD	8215(37.5)	9200(38.7)	10624(41.9)	11766(43.3)	12266(42.8)	13008(42.6)	15110(45.0)	12736(43.3)	13256(42.3)	13407(40.3)
Hypertension	16494(75.3)	17965(75.6)	19604(77.3)	21378(78.7)	22729(79.3)	24621(80.7)	27416(81.7)	23858(81.1)	25576(81.5)	27412(82.4)
Diabetes	6514(29.7)	7221(30.4)	7937(31.3)	8758(32.3)	9537(33.3)	10259(33.6)	12061(35.9)	10503(35.7)	11225(35.8)	11862(35.7)
Stage 3-5 CKD	704(3.2)	867(3.6)	1098(4.3)	1432(5.3)	1691(5.9)	2137(7.0)	2552(7.6)	2417(8.2)	2993(9.5)	3566(10.7)
Heart Failure	2739(12.5)	3053(12.9)	3268(12.9)	3488(12.8)	3789(13.2)	4256(13.9)	4931(14.7)	4390(14.9)	4975(15.9)	5224(15.7)

To examine the temporal trends of statin use and outcomes without the influence of the change in baseline characteristics, the main analyses applied a weight so patient characteristics were similar across different years as demonstrated in eTable 3.

© 2020 Yao X et al. *JAMA Network Open*.

eTable 3. Patient characteristics across different years with weight

Characteristics	2007	2008	2009	2010	2011	2012	2013	2014	2015	2016
**Age - yr**										
Median	66	66	66	67	67	67	67	67	67	67
Interquartile range	56-75	56-75	56-75	56-75	56-75	56-75	56-75	56-75	56-75	57-75
**Female sex –%**	46.3	46.0	46.1	46.1	46.0	46.2	46.3	46.4	46.4	46.4
**Race - %**										
Asian	3.3	3.0	3.3	3.3	3.3	3.4	3.5	3.5	3.4	3.4
Black	13.9	13.8	13.6	13.0	13.0	12.8	12.8	12.8	12.8	12.8
Hispanic	10.4	10.5	10.8	10.6	10.3	10.4	10.5	10.5	10.5	10.5
White	69.6	69.7	69.3	69.9	70.3	70.3	70.2	70.2	70.2	70.2
Other/unknown	2.8	3.0	3.0	3.1	3.0	3.0	3.0	3.0	3.0	3.0
**Index event – (%)**										
MI, Angina or Revascularization	36.5	36.9	36.6	36.9	36.9	36.8	36.6	36.6	36.6	36.6
Ischemic stroke or TIA	23.6	23.4	23.6	23.1	23.2	23.2	23.1	23.0	23.1	23.1
PAD	39.9	39.8	39.8	40.0	39.9	40.1	40.3	40.4	40.3	40.3
**Hypertension –%**	82.4	82.5	82.5	82.3	82.4	82.4	82.4	82.4	82.4	82.4
**Diabetes–%**	35.6	35.4	35.9	35.7	35.6	35.6	35.7	35.7	35.7	35.7
**Stage 3-5 CKD –%**	10.4	10.4	10.7	10.7	10.6	10.7	10.7	10.7	10.7	10.7
**Heart Failure–%**	16.4	16.3	16.0	16.0	15.9	15.7	15.7	15.7	15.7	15.7

© 2020 Yao X et al. *JAMA Network Open*.

eTable 4. Percentages of Patients Using Statins within 30 Days of Discharge With and Without Weights

	2007	2008	2009	2010	2011	2012	2013	2014	2015	2016
**With weights**										
Overall	50.3	51.2	53.6	55.0	56.7	57.0	58.7	59.1	59.8	59.9
MI, Angina or Revascularization	75.5	75.3	77.4	78.4	78.7	79.6	81.6	82.6	81.5	80.9
Ischemic stroke or TIA	45.1	45.4	49.8	52.1	56.2	57.5	58.9	61.6	63.3	65.8
PAD	30.3	32.3	34.0	35.0	36.7	36.0	37.8	36.5	38.0	37.5
**Without weights**										
Overall	52.2	52.5	52.8	53.5	55.3	55.7	57.0	57.9	59.0	59.9
MI, Angina or Revascularization	78.0	78.2	79.7	80.1	80.3	80.4	82.0	83.0	81.6	80.9
Ischemic stroke or TIA	42.8	43.4	47.7	50.1	55.0	56.5	58.8	61.4	63.1	65.8
PAD	28.2	30.0	31.1	32.7	34.3	34.5	37.5	36.0	37.9	37.5

P<0.001 for all trends

All the main analyses used a weight to balance the differences in baseline characteristics across years, and the results were very similar with or without the weight.

© 2020 Yao X et al. *JAMA Network Open*.

eTable 5. Percentages of Patients Using Statins within 90 Days of Discharge

	2007	2008	2009	2010	2011	2012	2013	2014	2015	2016
**Overall**	52.6	52.9	55.4	56.5	58.2	57.6	59.1	59.6	59.9	60.3
MI, Angina or Revascularization	78.2	77.7	79.5	80.1	80.4	80.5	82.7	83.3	81.9	82.1
Ischemic stroke or TIA	48.1	47.3	52.6	54.2	58.7	59.1	60.2	63.7	64.5	66.7
PAD	31.9	33.3	34.9	36.2	37.5	36.0	37.5	36.1	37.5	37.1

P<0.001 for all trends

© 2020 Yao X et al. *JAMA Network Open*.

eTable 6. Percentages of patients using high-intensity statins among patients who used statins within 30 days of discharge

	2007	2008	2009	2010	2011	2012	2013	2014	2015	2016
**Overall**	25.0	26.3	26.2	25.3	25.1	28.0	31.4	40.0	45.3	49.2
MI, Angina or Revascularization	32.0	33.0	32.3	30.9	31.3	36.7	43.0	55.5	60.5	66.0
Ischemic stroke or TIA	15.8	18.6	19.9	18.9	18.9	19.6	22.4	30.3	40.5	45.1
PAD	17.3	18.1	18.6	19.4	18.3	17.9	16.6	17.7	20.1	20.4

P<0.001 for all trends

© 2020 Yao X et al. *JAMA Network Open*.

eTable 7. Patient characteristics associated with high-intensity statin use

	% high-intensity statin	Adjusted Odds Ratio, 95% CI	P value
**Age**			
<65	39.1%	Reference	Reference
65-74	30.8%	0.77 (0.75, 0.79)	<0.001
≥75	23.5%	0.56 (0.54, 0.58)	<0.001
**Sex**			
Male	36.7%	Reference	Reference
Female	26.6%	0.79 (0.77, 0.81)	<0.001
**Race**			
Asian	27.7%	0.85 (0.80, 0.90)	<0.001
Black	32.3%	1.15 (1.11, 1.19)	<0.001
Hispanic	29.7%	0.93 (0.90, 0.97)	<0.001
White	33.2%	Reference	Reference
Other/unknown	32.6%	0.99 (0.93, 1.05)	0.74
**ASCVD Type**			
MI, Angina, or revascularization	42.4%	Reference	Reference
Ischemic stroke or TIA	26.0%	0.48 (0.47, 0.49)	<0.001
PAD	18.5%	0.32 (0.31, 0.33)	<0.001
**Hypertension**			
No	36.6%	Reference	Reference
Yes	32.0%	0.96 (0.93, 0.99)	0.02
**Diabetes**			
No	33.4%	Reference	Reference
Yes	31.3%	1.04 (1.02, 1.06)	0.001
**Stage 3-5 CKD**			
No	32.9%	Reference	Reference
Yes	29.9%	1.04 (1.01, 1.08)	0.02
**Heart failure**			
No	32.2%	Reference	Reference
Yes	34.1%	0.97 (0.95, 1.00)	0.07
**Index year**			
2007	25.0%	Reference	Reference
2008	26.3%	1.08 (1.03, 1.13)	0.003
2009	26.2%	1.09 (1.04, 1.15)	<0.001
2010	25.3%	1.06 (1.01, 1.11)	0.03
2011	25.1%	1.06 (1.01, 1.11)	0.03
2012	28.0%	1.23 (1.18, 1.30)	<0.001
2013	31.4%	1.48 (1.41, 1.55)	<0.001
2014	40.0%	2.21 (2.11, 2.31)	<0.001
2015	45.3%	2.82 (2.69, 2.96)	<0.001
2016	49.2%	3.38 (3.23, 3.54)	<0.001

© 2020 Yao X et al. *JAMA Network Open*.

eTable 8. Percentages of patients with PDC≥80% among patients who used statins within 30 days of discharge

	2007	2008	2009	2010	2011	2012	2013	2014	2015	2016
**Overall**	58.7	60.5	60.7	61.6	61.8	63.9	65.1	66.4	69.3	70.5
MI, Angina or Revascularization	61.2	64.1	65.3	65.0	66.0	67.4	69.2	71.5	73.4	74.6
Ischemic stroke or TIA	52.2	55.4	53.5	56.3	54.4	56.9	57.6	58.3	60.5	62.7
PAD	58.6	56.8	57.4	59.1	59.9	63.6	63.6	63.8	69.5	70.4

P<0.001 for all trends

© 2020 Yao X et al. *JAMA Network Open*.

eTable 9. Patient characteristics associated with adherence

	% PDC≥80%	Adjusted Odds Ratio, 95% CI	P value
**Age**			
<65	60.2%	Reference	Reference
65-74	66.6%	1.38 (1.34, 1.41)	<0.001
≥75	67.1%	1.47 (1.43, 1.52)	<0.001
**Sex**			
Male	65.8%	Reference	Reference
Female	61.7%	0.86 (0.84, 0.88)	<0.001
**Race**			
Asian	63.6%	0.86 (0.81, 0.92)	<0.001
Black	54.4%	0.61 (0.59, 0.64)	<0.001
Hispanic	54.5%	0.60 (0.58, 0.63)	<0.001
White	66.8%	Reference	Reference
Other/unknown	67.3%	0.99 (0.93, 1.06)	0.87
**ASCVD Type**			
MI, Angina, or revascularization	67.9%	Reference	Reference
Ischemic stroke or TIA	57.1%	0.62 (0.61, 0.64)	<0.001
PAD	62.6%	0.79 (0.77, 0.81)	<0.001
**Hypertension**			
No	62.7%	Reference	Reference
Yes	64.3%	1.06 (1.03, 1.10)	<0.001
**Diabetes**			
No	64.4%	Reference	Reference
Yes	63.7%	1.01 (0.99, 1.04)	0.37
**Stage 3-5 CKD**			
No	64.0%	Reference	Reference
Yes	65.3%	1.02 (0.98, 1.06)	0.29
**Heart failure**			
No	63.8%	Reference	Reference
Yes	65.3%	0.96 (0.93, 0.99)	0.004
**Index year**			
2007	58.7%	Reference	Reference
2008	60.5%	1.08 (1.03, 1.14)	0.002
2009	60.7%	1.10 (1.05, 1.16)	<0.001
2010	61.6%	1.14 (1.08, 1.20)	<0.001
2011	61.8%	1.15 (1.09, 1.20)	<0.001
2012	63.9%	1.26 (1.20, 1.33)	<0.001
2013	65.1%	1.33 (1.27, 1.40)	<0.001
2014	66.4%	1.42 (1.35, 1.49)	<0.001
2015	69.3%	1.62 (1.54, 1.70)	<0.001
2016	70.5%	1.72 (1.64, 1.81)	<0.001

© 2020 Yao X et al. *JAMA Network Open*.

eTable 10. Percentages of patients using a generic statin and cost per 30-day supply

	2007	2008	2009	2010	2011	2012	2013	2014	2015	2016
**Generic statin, %**	42.0	53.4	59.6	64.6	64.4	81.8	89.4	90.5	91.5	94.9
**Total cost**										
Mean±SD	78.1±50.7	66.7±54.4	59.5±56.2	55.6±59.1	62±67.9	49.4±110.4	32.3±51.4	28.5±53.9	29.7±59.6	25.2±56.1
Median	88.9	47.9	18.6	14.3	14.1	17.3	14.1	10.2	12.3	10.8
IQR	25.6-126.0	15.7-117.2	13.4-122.0	11.9-132.1	10.5-147.0	8.8-79.7	8.2-25.1	7.8-16.8	6.6-19.1	6.9-15.0
**Out-of-pocket cost**										
Mean±SD	22.9±20.9	20.1±20.3	17.6±20.5	16.7±21.5	17.4±23.1	15.5±23	10.4±16.5	9.5±17.2	8.7±23.3	7.7±20.7
Median	20.0	11.2	11.1	8.1	8.1	8.1	6.2	7.1	2.2	2.0
IQR	7.6-31.9	5.2-31.4	4.4-27.8	5.4-25.4	4.3-27.0	3.2-21.2	3.1-10.4	2.7-10.2	2.0-10.1	1.6-10.0

The costs were adjusted to reflect cost for a 30-day supply in 2016 dollar; p<0.001 for all trends

© 2020 Yao X et al. *JAMA Network Open*.

eTable 11. Cumulative risk of major adverse cardiac event at one year (%)

	2007	2008	2009	2010	2011	2012	2013	2014	2015	2016
**Overall**	8.9	8.6	8.5	8.4	7.6	7.1	6.8	6.7	6.7	6.5
MI, Angina or Revascularization	12.3	12.4	12.6	12.0	11.0	10.7	10.7	9.7	10.1	10.0
Ischemic stroke or TIA	9.0	8.4	8.6	7.8	7.4	6.5	6.0	6.8	6.7	6.0
PAD	5.6	5.1	4.8	5.3	4.6	4.0	3.9	3.9	3.6	3.7

P<0.001 for all trends

Major adverse cardiac event include myocardial infarction, ischemic stroke, and revascularization

© 2020 Yao X et al. *JAMA Network Open*.

eTable 12. Association between statin use within 30 days of discharge and MACE

	Hazard Ratio, 95% CI	P value
**Overall**	0.90 (0.86, 0.94)	<0.001
MI, Angina or Revascularization	0.87 (0.84, 0.91)	<0.001
Ischemic stroke or TIA	0.93 (0.88, 0.98)	0.01
PAD	0.86 (0.82, 0.90)	<0.001

The use of statin was associated with a lower risk of MACE in the overall cohort and in each of the three groups. The interaction p value was 0.12 between statin use and ASCVD type.

The model adjusted for age, sex, race, hypertension, diabetes, chronic kidney disease, heart failure and the index year.

© 2020 Yao X et al. *JAMA Network Open*.

eTable 13. Statin intolerance within one year (%)

	2007	2008	2009	2010	2011	2012	2013	2014	2015	2016	P value
**Overall**	4.0	4.0	3.6	4.2	5.4	4.6	4.3	4.6	4.9	5.1	<0.001
MI, Angina or Revascularization	6.9	6.5	5.8	6.9	8.1	7.3	7.4	7.8	8.2	8.1	<0.001
Ischemic stroke or TIA	3.3	3.6	3.1	4.1	5.6	4.7	4.4	5.0	5.5	6.9	<0.001
PAD	1.8	1.8	1.9	1.8	2.7	2.1	1.6	1.5	1.7	1.6	0.01

P<0.001 for all trends

Statin intolerance was assessed during the first year after the ASCVD event, defined using an established algorithm which includes: (1) down-titration of statin dose or switch to a lower-intensity statin, or (2) initiation of ezetimibe or a PCSK9 inhibitor within 7 days before or any time after or discontinuing statins, or (3) diagnosis for rhabdomyolysis or adverse effect of an antihyperlipidemic agent, or (4) fills for ≥3 different statins.

© 2020 Yao X et al. *JAMA Network Open*.

eTable 14. Trends of statin use within 30 days of discharge (%), stratified by ASCVD type and age

	2007	2008	2009	2010	2011	2012	2013	2014	2015	2016	P value
**Overall**											
Age<65	51.5	52.5	53.7	54.3	54.9	55.0	57.5	58.0	58.2	57.6	<0.001
Age 65-74	55.0	56.5	58.8	59.2	61.3	62.6	62.9	63.2	64.5	64.5	<0.001
Age≥75	42.9	43.0	47.6	51.2	54.5	54.0	56.0	56.3	56.9	58.5	<0.001
**MI, Angina or Revascularization**											
Age<65	78.8	80.1	81.5	82.2	82.3	83.2	84.7	85.9	84.0	83.7	<0.001
Age 65-74	75.9	74.9	77.4	78.7	78.7	80.0	81.3	82.7	82.8	80.6	<0.001
Age≥75	66.8	65.2	68.1	70.4	71.8	71.6	75.1	74.9	74.1	75.3	<0.001
**Ischemic stroke or TIA**											
Age<65	42.5	43.0	46.1	47.3	52.2	52.6	53.7	55.7	57.8	60.1	<0.001
Age 65-74	52.5	53.4	56.7	58.0	61.3	63.5	65.2	69.0	70.1	73.3	<0.001
Age≥75	41.9	41.6	48.2	53.9	57.1	59.1	61.2	63.6	65.1	66.7	<0.001
**PAD**											
Age<65	26.8	29.2	28.8	29.6	28.5	27.7	28.2	27.3	28.0	27.5	0.16
Age 65-74	36.5	39.8	42.0	41.7	45.2	45.0	45.9	43.5	45.9	45.7	<0.001
Age≥75	28.7	29.1	33.2	35.3	39.7	38.6	41.4	40.9	42.0	42.4	<0.001

© 2020 Yao X et al. *JAMA Network Open*.

eTable 15. Trends of high-intensity statin use within 30 days of discharge (%), stratified by ASCVD type and age

	2007	2008	2009	2010	2011	2012	2013	2014	2015	2016	P value
**Overall**											
Age<65	28.8	31.4	31.5	30.5	30.5	34.0	38.7	48.1	54.5	59.4	<0.001
Age 65-74	24.9	24.6	25.0	23.0	23.8	26.5	29.5	37.9	43.3	46.1	<0.001
Age≥75	17.6	18.5	17.8	19.4	17.8	19.8	21.4	29.1	32.3	36.6	<0.001
**MI, Angina or Revascularization**											
Age<65	34.8	37.7	37.8	36.9	37.3	43.1	49.6	61.2	66.6	73.5	<0.001
Age 65-74	32.3	30.9	29.7	26.8	29.3	32.5	40.2	52.5	58.7	62.7	<0.001
Age≥75	23.2	23.6	22.0	22.7	20.7	28.1	30.6	45.2	47.2	53.0	<0.001
**Ischemic stroke or TIA**											
Age<65	16.7	21.7	22.4	19.5	20.5	21.9	22.9	31.9	44.9	49.9	<0.001
Age 65-74	17.2	16.7	21.0	18.9	19.1	22.2	23.9	31.9	41.5	45.5	<0.001
Age≥75	12.9	16.5	15.7	18.3	16.5	13.7	19.9	26.2	33.0	38.0	<0.001
**PAD**											
Age<65	21.1	21.5	21.4	22.9	21.0	19.9	21.4	22.7	25.1	25.5	0.001
Age 65-74	16.4	18.5	19.7	19.0	18.6	19.4	17.2	19.0	20.9	20.9	0.005
Age≥75	13.6	12.5	13.8	15.5	15.0	14.2	11.9	12.3	15.2	15.4	0.38

© 2020 Yao X et al. *JAMA Network Open*.

eTable 16. Trends of one-year statin adherence (%), stratified by ASCVD type and age

	2007	2008	2009	2010	2011	2012	2013	2014	2015	2016	P value
**Overall**											
Age<65	55.3	58.3	57.6	58.4	57.5	59.8	60.9	63.3	65.3	64.8	<0.001
Age 65-74	61.3	62.4	61.6	63.6	64.8	66.8	68.3	68.5	71.2	73.7	<0.001
Age≥75	61.2	61.7	64.8	64.4	64.5	66.5	67.7	68.7	72.7	74.3	<0.001
**MI, Angina or Revascularization**											
Age<65	60.5	63.6	63.7	65.0	64.2	66.2	68.2	70.9	72.6	71.9	<0.001
Age 65-74	61.7	63.6	64.5	64.1	69.0	70.3	71.0	72.2	74.0	77.7	<0.001
Age≥75	62.0	66.1	70.4	66.3	65.8	65.4	69.0	71.6	74.4	75.6	<0.001
**Ischemic stroke or TIA**											
Age<65	43.3	48.0	44.1	48.0	45.2	47.4	47.8	50.1	50.0	51.4	<0.001
Age 65-74	59.3	62.9	59.0	62.0	60.2	60.8	63.7	62.4	65.6	68.2	<0.001
Age≥75	56.0	56.8	58.5	60.8	59.3	64.5	64.7	64.5	68.1	69.8	<0.001
**PAD**											
Age<65	49.7	51.7	51.6	49.1	50.6	54.7	52.0	53.0	60.1	58.6	<0.001
Age 65-74	62.0	59.6	58.5	63.7	61.5	65.5	67.5	67.3	71.3	72.4	<0.001
Age≥75	64.8	59.6	62.6	65.0	67.1	69.7	68.4	68.3	74.3	76.4	<0.001

© 2020 Yao X et al. *JAMA Network Open*.

eTable 17. Trends of one-year risk of MACE (%), stratified by ASCVD type and age

	2007	2008	2009	2010	2011	2012	2013	2014	2015	2016	P value
**Overall**											
Age<65	6.3	5.7	5.7	5.5	5.3	4.9	4.9	5.1	5.2	5.0	<0.001
Age 65-74	9.4	8.7	9.1	8.8	7.4	7.2	7.1	6.4	7.0	6.4	<0.001
Age≥75	12.2	12.7	12.2	12.5	11.4	10.3	9.5	9.4	8.7	9.0	<0.001
**MI, Angina or Revascularization**											
Age<65	10.2	9.9	9.5	9.5	9.6	8.8	8.2	8.6	8.6	8.4	<0.001
Age 65-74	12.4	12.6	13.2	12.5	9.8	10.7	11.0	8.8	10.1	9.6	<0.001
Age≥75	17.0	17.4	18.0	16.3	15.3	14.5	15.6	13.5	13.2	13.7	<0.001
**Ischemic stroke or TIA**											
Age<65	5.7	4.8	4.9	4.9	3.6	3.8	3.9	4.4	4.2	3.7	<0.001
Age 65-74	10.1	8.2	10.8	7.8	7.9	8.0	6.9	7.8	8.2	6.0	<0.001
Age≥75	12.5	13.1	11.1	11.9	12.2	9.1	8.7	9.6	9.0	9.3	<0.001
**PAD**											
Age<65	2.4	1.9	2.2	1.7	2.0	1.7	1.9	1.6	1.8	2.0	0.09
Age 65-74	6.2	5.0	4.3	5.7	4.8	3.4	3.9	3.7	3.7	3.7	<0.001
Age≥75	8.9	9.3	8.8	10.1	7.9	7.9	6.1	6.7	5.7	5.8	<0.001

© 2020 Yao X et al. *JAMA Network Open*.

eTable 18. Trends of statin use within 30 days of discharge (%), stratified by ASCVD type and sex

	2007	2008	2009	2010	2011	2012	2013	2014	2015	2016	P value
**Overall**											
Male	56.6	57.4	59.3	59.8	61.7	62.3	64.1	64.6	65.6	65.2	<0.001
Female	43.0	43.9	47.0	49.3	50.9	50.9	52.4	52.8	53.0	53.9	<0.001
**MI, Angina or Revascularization**											
Male	77.3	78.0	79.4	79.8	80.3	81.1	83.8	84.7	83.8	83.7	<0.001
Female	71.8	69.6	73.2	75.7	75.5	76.5	77.1	78.0	76.8	75.4	<0.001
**Ischemic stroke or TIA**											
Male	48.3	48.7	51.1	53.5	58.4	59.7	61.6	64.0	66.0	69.2	<0.001
Female	42.3	42.4	48.6	50.8	54.3	55.6	56.6	59.6	60.8	62.7	<0.001
**PAD**											
Male	33.2	34.3	36.3	36.5	37.8	38.0	39.1	37.7	40.5	38.8	<0.001
Female	28.0	30.7	32.2	33.7	35.8	34.3	36.7	35.5	35.9	36.5	<0.001

© 2020 Yao X et al. *JAMA Network Open*.

eTable 19. Trends of high-intensity statin use within 30 days of discharge (%), stratified by ASCVD type and sex

	2007	2008	2009	2010	2011	2012	2013	2014	2015	2016	P value
**Overall**											
Male	27.8	29.6	29.2	28.1	28.3	32.0	36.1	45.3	51.0	55.8	<0.001
Female	20.7	21.2	21.7	21.5	20.5	22.2	24.7	32.5	37.1	40.0	<0.001
**MI, Angina or Revascularization**											
Male	33.3	34.6	34.5	33.0	33.7	39.4	45.7	57.8	63.6	69.4	<0.001
Female	28.8	29.1	27.3	26.3	26.0	30.8	37.1	50.3	53.4	58.7	<0.001
**Ischemic stroke or TIA**											
Male	17.5	21.5	21.0	20.8	20.5	21.1	24.8	32.6	43.1	50.1	<0.001
Female	14.1	15.8	18.9	17.2	17.4	18.3	20.2	28.3	38.0	40.3	<0.001
**PAD**											
Male	19.3	20.7	20.2	19.8	20.0	20.7	18.7	19.8	22.9	23.5	0.001
Female	15.3	15.6	17.2	19.1	16.8	15.4	14.7	15.9	17.6	17.6	0.38

© 2020 Yao X et al. *JAMA Network Open*.

eTable 20. Trends of one-year statin adherence (%), stratified by ASCVD type and sex

	2007	2008	2009	2010	2011	2012	2013	2014	2015	2016	P value
**Overall**											
Male	60.0	62.9	62.2	63.8	63.5	65.4	66.5	67.8	71.2	72.3	<0.001
Female	56.8	56.9	58.6	58.6	59.4	62.0	63.1	64.4	66.6	68.1	<0.001
**MI, Angina or Revascularization**											
Male	62.7	65.5	66.0	66.5	67.6	68.8	69.8	72.6	75.5	76.3	<0.001
Female	57.6	60.8	63.7	61.8	62.6	64.2	67.8	68.8	68.8	70.9	<0.001
**Ischemic stroke or TIA**											
Male	51.9	59.0	54.7	56.8	54.4	56.0	57.5	57.7	60.4	62.6	<0.001
Female	52.5	51.9	52.4	55.7	54.3	57.7	57.8	58.8	60.7	62.8	<0.001
**PAD**											
Male	57.9	57.7	57.1	61.5	59.8	64.0	65.1	62.8	69.7	71.1	<0.001
Female	59.3	56.0	57.7	57.0	60.0	63.2	62.4	64.6	69.4	69.8	<0.001

© 2020 Yao X et al. *JAMA Network Open*.

eTable 21. Trends of one-year risk of MACE (%), stratified by ASCVD type and sex

	2007	2008	2009	2010	2011	2012	2013	2014	2015	2016	P value
**Overall**											
Male	9.5	9.2	9.2	8.9	8.3	7.7	7.6	7.4	7.5	7.1	<0.001
Female	8.1	7.8	7.7	7.8	6.8	6.3	6.0	5.9	5.8	5.9	<0.001
**MI, Angina or Revascularization**											
Male	11.5	12.1	12.0	11.5	10.7	10.0	10.3	9.3	10.2	9.7	<0.001
Female	14.1	13.2	13.7	13.1	11.8	12.1	11.5	10.6	9.8	10.6	<0.001
**Ischemic stroke or TIA**											
Male	9.5	8.4	9.3	8.1	7.8	6.8	6.3	7.5	7.1	6.1	<0.001
Female	8.7	8.4	7.9	7.5	7.1	6.3	5.8	6.2	6.4	5.9	<0.001
**PAD**											
Male	6.7	5.9	5.3	5.7	5.2	5.0	4.7	4.7	4.0	4.4	<0.001
Female	4.7	4.4	4.4	5.0	4.1	3.2	3.2	3.2	3.3	3.2	<0.001

© 2020 Yao X et al. *JAMA Network Open*.

eFigure 1. Patient selection flow diagram

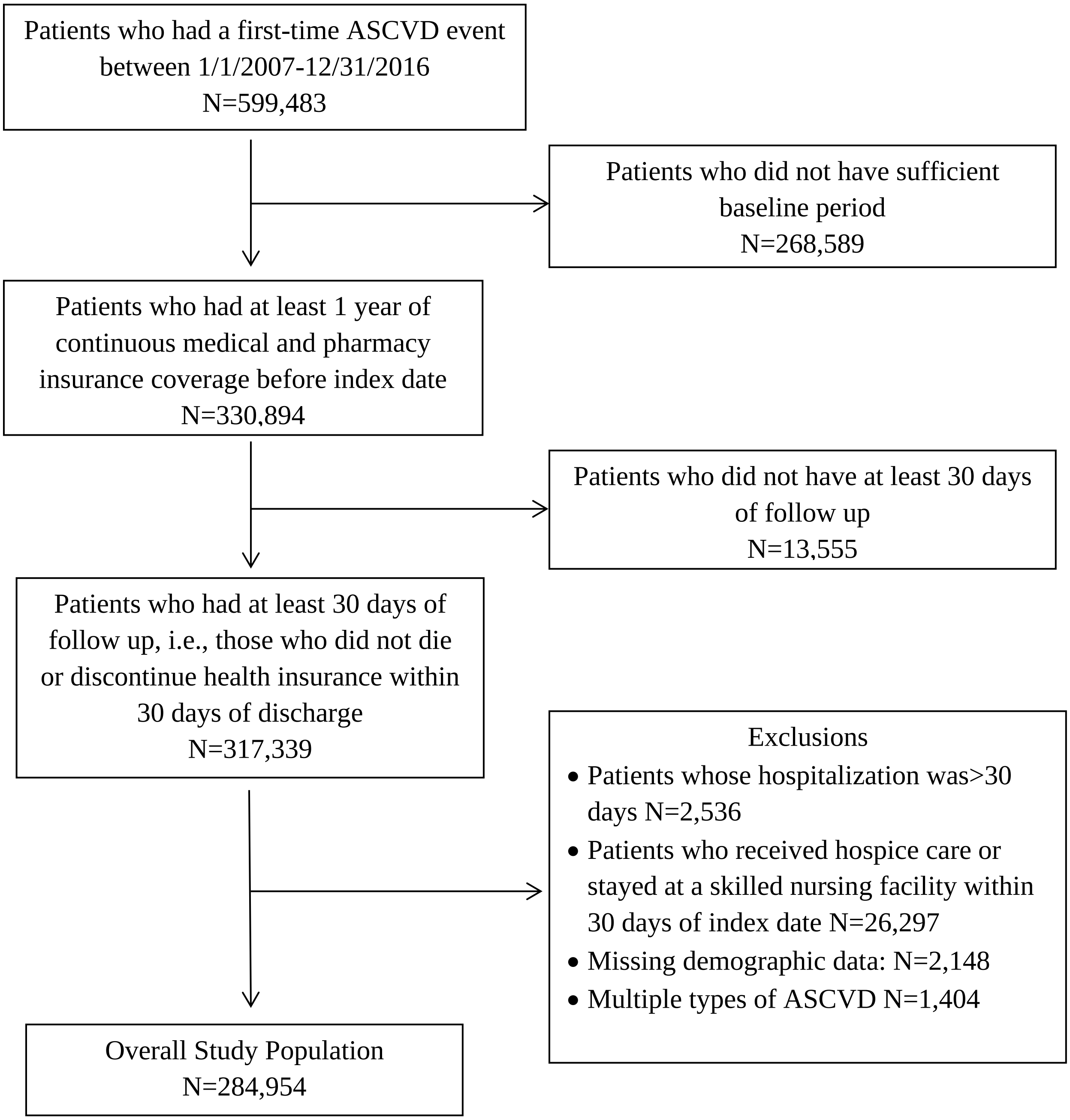

© 2020 Yao X et al. *JAMA Network Open*.

eFigure 2. One-year cumulative risk of lower extremity vascular complications among patients with PAD

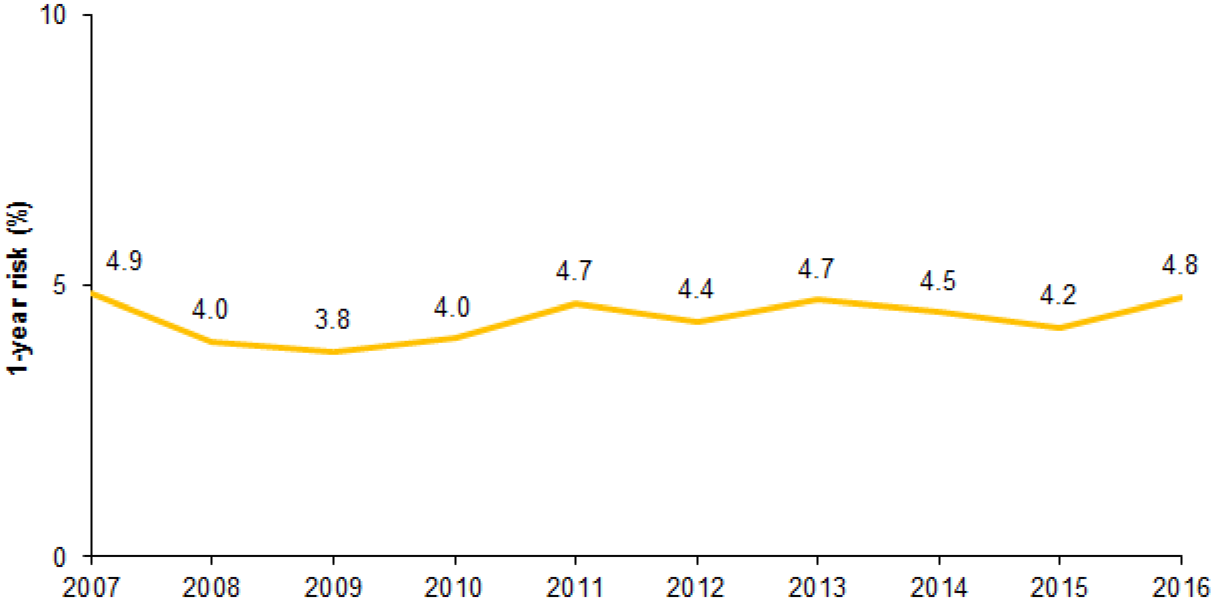

Lower extremity vascular complications include surgical or endovascular procedures and amputations.

© 2020 Yao X et al. *JAMA Network Open*.

eFigure 3. Trends of Statin Use, Stratified by ASCVD Type and Age

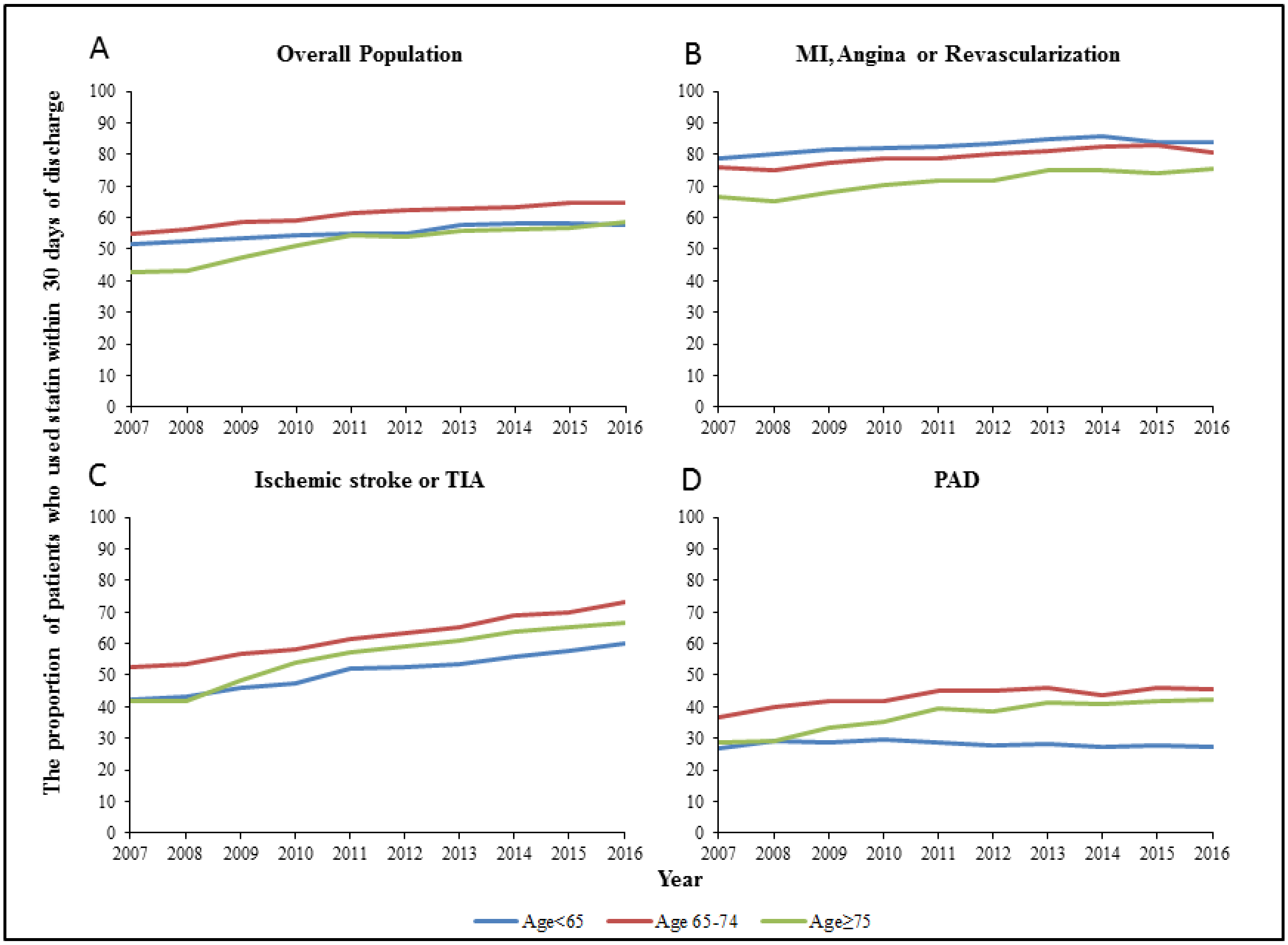

The proportion of patients who used statin within 30 days of discharge in the overall population (Panel A), patients with MI, angina or revascularization (Panel B), ischemic stroke or TIA (Panel C), and PAD (panel D).

© 2020 Yao X et al. *JAMA Network Open*.

eFigure 4. Trends of Statin Use, Stratified by ASCVD Type and Sex

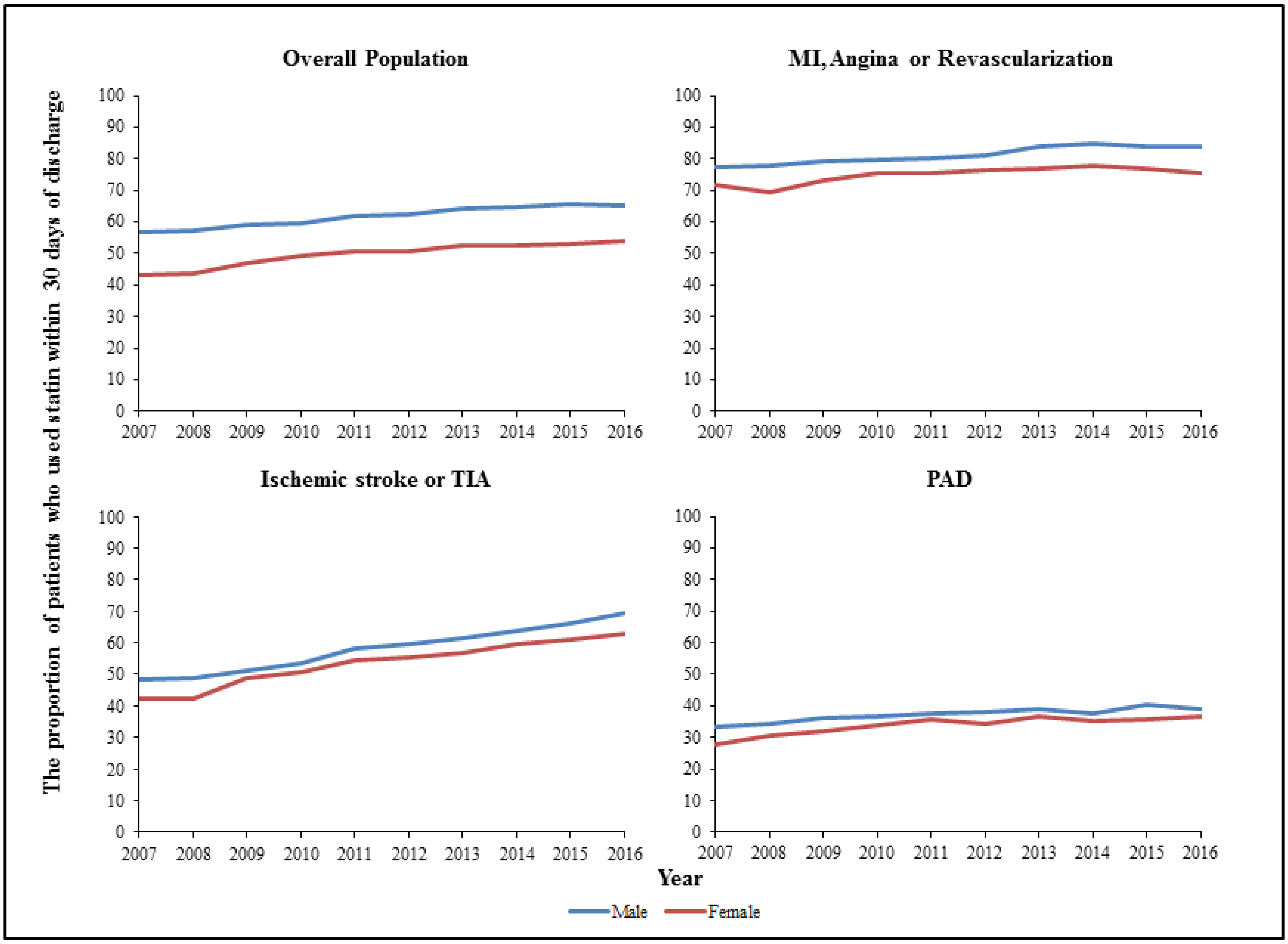

The proportion of patients who used statin within 30 days of discharge in the overall population (Panel A), patients with MI, angina or revascularization (Panel B), ischemic stroke or TIA (Panel C), and PAD (panel D).

© 2020 Yao X et al. *JAMA Network Open*.

eFigure 5. Trends in one-year risk of MACE (%), stratified by ASCVD type and Age

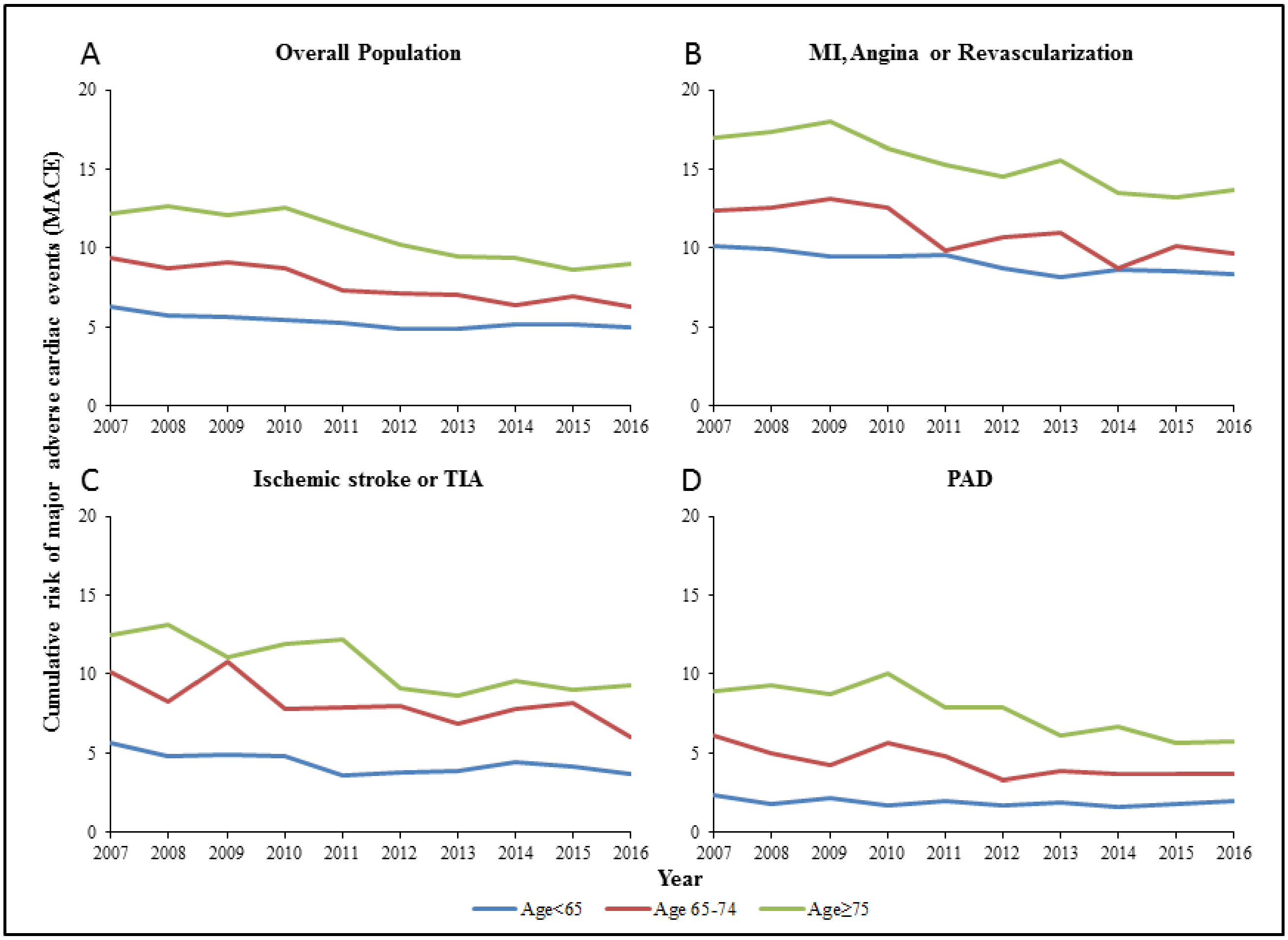

Cumulative risk of major adverse cardiac events (MACE) at 1 year, including myocardial infarction, ischemic stroke, revascularization, and all-cause mortality in the overall population (Panel A), patients with MI, angina or revascularization (Panel B), ischemic stroke or TIA (Panel C), and PAD (panel D).

© 2020 Yao X et al. *JAMA Network Open*.

eFigure 6. Trends in one-year risk of MACE (%), stratified by ASCVD type and sex

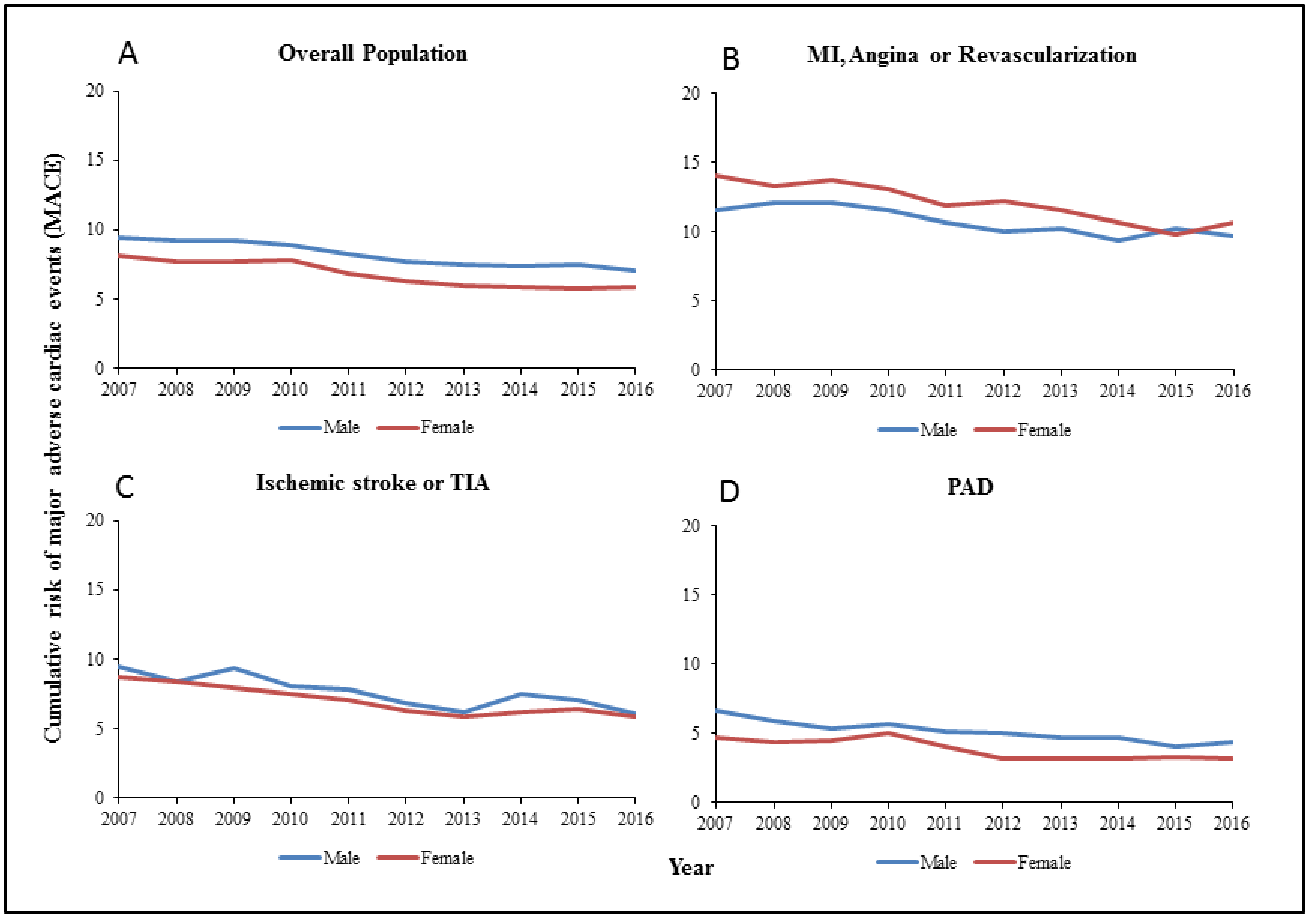

Cumulative risk of major adverse cardiac events (MACE) at 1 year, including myocardial infarction, ischemic stroke, revascularization, and all-cause mortality in the overall population (Panel A), patients with MI, angina or revascularization (Panel B), ischemic stroke or TIA (Panel C), and PAD (panel D).

© 2020 Yao X et al. *JAMA Network Open*.

eAppendix. Statistical Analysis Plan

Statistical Analysis Plan Date: 6/5/2018

The purpose of this analysis plan is to provide guide to analysts when conducting the study. Most of the content will be included in the manuscript in order to guide researchers who want to replicate our findings or conduct similar studies. We also provided justifications for our methods and decisions so other researchers can make a choice or adjust their methods accordingly.

ABBREVIATIONS

ASCVD	Atherosclerotic cardiovascular disease
CI	Confidence interval
HR	Hazard ratio
IQR	Interquartile range
MACE	Major adverse cardiac events
MI	Myocardial infarction
PAD	Peripheral artery disease
PCSK9	Proprotein convertase subtilisin–kexin type 9
TIA	Transient ischemic attack

© 2020 Yao X et al. *JAMA Network Open*.

Key Definition

Index Date (variable name index_date) the date of a patient's first ASCVD event. If a patient was hospitalized, the index date will be the discharge date from the hospital. Please see page for more details.

Baseline Period (variable name baseline)

Any time before the index date, used to establish a patient's medical history. Detailed description and justification can be found on page 12.

Study Period

The study population will be patients who had an ASCVD event between 1/1/2007-12/31/2016. Patients can be follow up until 12/31/2017, which is the most recent data we will have at the time of the analysis. Please see page 19 for more details.

© 2020 Yao X et al. *JAMA Network Open*.

1. BACKGROUND AND OBJECTIVES

Atherosclerotic cardiovascular disease (ASCVD) is highly prevalent, affecting approximately 20 million people in the United States.[1, 2] Statin therapy is a key pillar of secondary prevention for these patients,[3] but numerous studies showed a significant rate of non-adherence and under-treatment.[4, 5] As the benefits of adhering to statin therapy being repeatedly demonstrated,[6-8] the past decade has witnessed extensive efforts and innovative interventions to improve statin adherence.[9] During this time period, guidelines have been changed regarding high-intensity statins[10] and generic high-intensity statins have become widely available.

However, there is a lack of evidence documenting population trends of statin use, adherence, and outcomes. Previous studies of statin use focused on patients with myocardial infarction (MI), who tend to have better use of guideline-recommended statin therapy; few studies have examined trends of statin use in patients with other types of ASCVD, such as ischemic stroke or peripheral artery disease (PAD).[1, 11, 12] Furthermore, although an increase in the statin use and intensity should lead to a decrease in major adverse cardiac events (MACE), few data existed to demonstrate the population trend of MACE in patients with ASCVD.

As such, the current study will use a large national cohort of patients with all types of ASCVD managed at diverse routine practice settings to examine the use, adherence, cost, and outcomes of statin therapy for secondary prevention between 2007 and 2016. Specifically, the goal is to assess whether there have been improvements in treatment and outcomes and to identify gaps to guide future quality improvement efforts.

© 2020 Yao X et al. *JAMA Network Open*.

2. STUDY DESIGN AND DATA SOURCE

We will conduct a retrospective cohort analysis using OptumLabs Data Warehouse, which contains over 130 million privately insured and Medicare Advantage enrollees of all ages and races from all 50 states.[13, 14] In 2014, this amounted to 19% of all commercially insured and Medicare Advantage beneficiaries in the U.S.

OptumLabs includes patients of all ages and races from all 50 states. To demonstrate the similarities between the OptumLabs cohort and a nationally representative cohort, we used 4,991,144 people aged ≥40 years from the OptumLabs cohort in 2013 and compared them with a nationally representative cohort aged ≥40 years in a previous study using the Medical Expenditure Panel Survey (MEPS) 2012-2013 data.[1]

Table 1. Comparison of OptumLabs Data and a US Nationally Representative Cohort

	OptumLabs Cohort	Nationally Representative Cohort
**Age, mean, y**	58.3	58.5
**Age category, y**		
<65	69.4	69.9
65-74	18.4	17.4
≥75	12.1	12.7
**Sex**		
Male	47.6	47.8
Female	52.4	52.2
**Race/ethnicity**		
Asian	3.9	4.9
Black	10.6	10.8
Hispanic	8.4	11.4
White	74.5	71.1
Other/unknown	2.7	1.8

© 2020 Yao X et al. *JAMA Network Open*.

3. STUDY POPULATION

We will identify adult patients (age ≥21 years) who had their first ASCVD event between 1/1/2007-12/31/2016. The date of a patient's first ASCVD event will be defined as the index date. If a patient was hospitalized, the index date will be the discharge date from the hospital. Patients will be required to have continuous medical and pharmacy insurance coverage for at least one year before the index date, to allow sufficient data to capture a relatively complete medical history. The time before the index date will be defined as the baseline. Based on our prior studies, patients on average have about 3-4 years of baseline period, which is stable over time, especially after adjusting for patient characteristics (e.g., age). This method of utilizing all baseline data available has been used in our previous studies.[15] Only patients who did not have ASCVD any time during the baseline period will be included.

ASCVD will be defined as MI, angina, coronary revascularization, ischemic stroke, TIA, or PAD. This definition is based on the 2013 American College of Cardiology/American Heart Association (ACC/AHA) guidelines.[10] We will identify MI, angina, ischemic stroke, and TIA, based on the presence of a primary diagnosis on an emergency room visit or a hospitalization. Angina patients will be required to have additional diagnosis codes indicating the presence of coronary artery disease (ICD-9 411.x, 414.x except 414.10 and 414.19; ICD-10 I24.x, I25.x except I25.3) at any position in either inpatient or outpatient claims at baseline (including the index date), in order to avoid the inclusion of patients with non-cardiac chest pain that resulted in the use of an angina code. We will exclude ischemic stroke or TIA patients who had a diagnosis of atrial fibrillation (ICD-9 427.31, ICD-10 I48.0, I48.1, I48.2, and I48.91) at baseline or on the index date, in order to limit to ischemic stroke or TIA presumed to be of atherosclerotic origin.[16] Coronary revascularization will be identified using procedure codes. PAD will be identified

© 2020 Yao X et al. *JAMA Network Open*.

using a validated algorithm with positive predictive value (PPV) of 91%.[17] In the validation study, PAD was defined as atherosclerotic occlusive arterial disease of the lower extremities, including arteries distal to the aortic bifurcation. The vascular laboratory diagnostic criteria for PAD were: (1) a resting/post-exercise ABI $\leq$0.9; or (2) the presence of poorly compressible arteries (ABI >1.4; or ankle blood pressure >255 mm Hg).

We will exclude patients who died or discontinued health insurance within the 30 days of discharge, patients whose hospitalization at the index date was more than 30 days, patients who received hospice care or stayed at a skilled nursing facility within 30 days of the index date, and patients who had invalid or missing demographic data.

The Mayo Clinic Institutional Review Board (IRB) exempted this study from review, because the study used pre-existing, de-identified data.

© 2020 Yao X et al. *JAMA Network Open*.

Table 1. Diagnosis and Procedure Codes to Identify ASCVD Events

	Diagnosis Codes		Procedure Codes		
	**ICD-9-CM**	**ICD-10-CM**	**CPT**	**ICD-9**	**ICD-10**
**Myocardial infarction**	410.x1	I21.x			
**Angina**	413.x	I20.x, I23.7, I25.11x, I25.7x			
**CABG**	414.02, 414.03, 414.04 414.05, V45.81	Z95.1, T82.21x, I25.7x (exclude I25.75), I25.810, I25.812	33510-33519, 33521-33523, 33533-33536 4110F	36.1x	02100xx, 02110xx, 02120xx, 02130xx
**PCI**	V45.82	Z98.61, Z95.5	92920-92929, 92933-92938, 92941-92944, 92980-92984, 92995, 92996, 92975, 92977	36.0x, 00.66	0270xxx, 0271xxx, 0272xxx, 0273xxx, 02C0xxx, 02C1xxx, 02C2xxx, 02C3xxx
**Ischemic stroke**	433.x1, 434.x1, 436	I63.x			
**Transient ischemic attack**	435.x	G45.x			

We will use a broader set of codes to exclude those with a history of ASCVD at baseline – we will use 410.x, 411.0, 412, I22-I23, I24.1, and I25.2 for a history of MI and I69.3x for a history ischemic stroke, in addition to the codes in the above table. The diagnosis codes for CABG and PCI will be used for exclusion of patients with prior events only (i.e., they won't be used to identify a new event).

© 2020 Yao X et al. *JAMA Network Open*.

4. MEASUREMENTS

4.1 Baseline Characteristics

Baseline characteristics include socio-demographic characteristics, and medical history. Socio-demographic characteristics include age, sex, and race, determined at the time of index date. Race/ethnicity is provided by OptumLabs, classified as non-Hispanic White (White), non-Hispanic Black (Black), Asian, Hispanic, or other/unknown. Self-report was the primary source, and when it was missing, imputation was made by the data provider based on other available administrative data.[18] Medical history will be determined using patients' physician, facility and pharmacy claims before the index date. All the comorbidities (except diabetes) will be defined as a diagnosis code or procedure code at any position in either inpatient or outpatient claims at baseline. Diabetes will be defined using an established algorithm, i.e., at least 2 outpatient encounters, or 1 inpatient encounter, or a antidiabetic prescription (not including metformin).[19] We will not use antihypertensive drugs to define hypertension, since some antihypertensive drugs can be used to treat other conditions.

© 2020 Yao X et al. *JAMA Network Open*.

Table 2. Diagnosis and Procedure Codes to Identify ASCVD Events

	Diagnosis Codes		Procedure Codes		
**Comorbidities**	**ICD-9**	**ICD-10**	**CPT**	**ICD-9**	**ICD-10**
**Hypertension**	401-405	I10-I15			
**Diabetes**	250.x, 362.0x, 357.2, 366.41	E10.x, E11.x, E13.x			
**CKD (stage 3-5)**	585.3, 585.4, 585.5, 585.6, 403.01, 403.11, 403.91, 404.02, 404.03, 404.12, 404.13, 404.92, 404.93, 792.5, 996.81, V42.0, V45.1, V45.11, V45.12, V56.x, V56.0, V56.1, V56.2, V56.3, V56.31, V56.32, V56.8	N183, N184, N185, N186, I12.0, I13.11, I13.2, I95.3, R88.0, T81.502x, T81.512x, T81.522x, T81.532x, T81.592x, T85.611x, T85.621x, T85.631x, T85.651x, T85.71x, T86.1x, Y84.1, Z48.22, Z49.x, Z91.15, Z94.0, Z99.2	50340, 50360, 50365, 50370, 90935, 90937, 90940, 90945, 90947, 90957, 90958, 90959, 90960, 90961, 90962, 90965, 90966, 90969, 90970, 90999, G0257, S9335, 99512	39.95, 54.98, 55.53, 55.6, 55.69	0TT00ZZ, 0TT04ZZ, 0TT10ZZ, 0TT14ZZ, 0TT30ZZ, 0TT34ZZ, 0TT37ZZ, 0TT38ZZ, 0TT40ZZ, 0TT44ZZ, 0TY00Z0, 0TY00Z1, 0TY00Z2, 0TY10Z0, 0TY10Z1, 0TY10Z2, 3E1M39Z, 5A1D00Z, 5A1D60Z
**Heat failure**	428.x, 402.x1, 404.x1, 404.x3, 398.91	I50.x, I97.13x, I11.0, I13.0, I13.2, I09.81			
**Smoking**	305.1, 649.0x, 989.84, V15.82	F17.x, O99.33x, T65.2x, Z53.01, Z71.6, Z72.0, Z87.891	1034F, 4000F, 4001F, 4004F,99406, 99407, C9801, C9802, D1320, G0375, G0376, G0436, G0437, G8402, G8453, G8455, G9276, G9458, G9792		
**Depression**	290.13, 290.21, 290.43, 296.2x, 296.3x, 296.5x, 296.82, 298.0, 300.4, 301.12, 305.8x, 309.0, 309.1, 309.28, 311, 969.0x, E854.0, E939.0	F19.1x, F31.3x, F31.4, F31.5, F31.75, F31.76, F32.x, F33.x, F34.1, F43.21, F43.23, T43.0x, T43.1x, T43.2x			

© 2020 Yao X et al. *JAMA Network Open*.

4.2 Follow up

The end of the study period will be December 31, 2017. Patients will be followed until December 31, 2017, or the date of disenrollment in health insurance plans or death, whichever happened first.

4.3 Post-Discharge Statin Use

Post-discharge statin use will be defined as the use of statin within 30 days of discharge. The use of statin will be determined by the prescription fill in pharmacy claims. If a patient filled a statin prescription prior to the index date, and there were residual pills left (determined based on days supplied), the patient will be considered as on statin as well. As a sensitivity analysis, we will test the prescription fill within 90 days of discharge without considering prescription before the index date. We will calculate the proportion of patients who used prescription within 30 days of discharge. We will also calculate the proportion of statin users who received a high-intensity statin, defined as atorvastatin 40 or 80 mg, rosuvastatin 20 or 40 mg, and simvastatin 80 mg.[10] The FDA issued a warning against the use of simvastatin 80 mg in 2011, however, we will include this drug in the study since it was used early in the study period. As a sensitivity analysis, we will consider patients who received moderate-intensity statins plus ezetimibe in addition to high-intensity statins as high-intensity lipid-lowering treatment.

There is concern that whether the generic drug discount programs (e.g., Walmart's $4 drug plan) will affect the completeness of prescription information in the claims database. However, in this study, the generic drug discount programs would have little impact on the completeness of the data. Frist, statins offered by drug discount programs are very limited. For example, Walmart and CVS only offer lovastatin 10 mg and 20 mg, which are low-intensity

© 2020 Yao X et al. *JAMA Network Open*.

statins, but in patients with ASCVD, high-intensity statins (or moderate-intensity statin in patients with age>75 or safety concerns) are recommended.[10] Furthermore, lovastatin, regardless intensity, is not commonly used. In a previous study, only 10% of statin users received lovastatin.[12] Second, in patients with insurance, the out-of-pocket cost for a generic statin is very low – the median was $2 for a 30-day supply in our dataset. Therefore, they may not need to turn to the $4 drug plan. Third, some previous studies assessing the completeness of claims data suggested that the payers have captured the claims when patients used a generic drug discount program.[20, 21]

4.4 Statin Adherence

We will assess patients' adherence at one year, measured by the proportion of days covered (PDC). The analysis will be limited to patients who used statin within the first 30 days post discharge and continuously enrolled in health insurance during the 12 months after the index date. Since some patients were re-hospitalized during follow up, the number of days in a hospital will be deducted from the denominator when calculating PDC. Patients with a PDC≥80% will be considered as being adherent to statin therapy. When calculating PDC, all statins will be counted, even if the drug or the dose is different than what patients first received.

4.5 Statin Cost

Among patients who used statin within 30 days of discharge, we will calculate the cost of the statin therapy, standardized toward a 30-day supply (i.e., cost*30/days supplied). We will also adjust the cots to reflect the 2016 dollars using the Gross Domestic Product (GDP) price index, as recommended by the Agency for Healthcare Research and Quality (AHRQ).[22, 23] We will calculate the amount paid by health plans, amount paid by patients, and the overall cost.

© 2020 Yao X et al. *JAMA Network Open*.

4.6 Cardiovascular Outcomes

We will assess the major adverse cardiac events (MACE), including MI, ischemic stroke, revascularization, and all-cause mortality during follow up. MI, ischemic stroke and revascularization will be defined the same way as how we identified the index events. As a sensitivity analysis, we will examine the lower extremity vascular complications, including surgical or endovascular procedures and amputations, in patients with PAD.

4.7 Statin Intolerance

The statin intolerance will be examined within one year after the index date. The analysis will be limited to patients who had continuous insurance coverage health insurance during the 12 months. The definition of statin intolerance is based on an established algorithm,[24, 25] including

1. Down-titration of statin dose or switch to a lower-intensity statin; or

2. Initiation of ezetimibe or a PCSK9 inhibitor within 7 days before or any time after or discontinuing statins; or

3. Diagnosis for rhabdomyolysis (ICD-9 728.88 or ICD-10 M62.82) or “adverse effect of an antihyperlipidemic agent” (ICD-9 E942.2 or ICD-10 T46.6x) at any position on an inpatient or outpatient claim; or

4. Fills for ≥3 different statins.

© 2020 Yao X et al. *JAMA Network Open*.

4.8 Internal Validation of OLDW Data

The codes and algorithms used herein have been commonly used and validated in many previous studies.[26-32] We also leveraged the ability to link to laboratory results and electronic health records to validate our diagnosis codes. For example, we compared eGFR with the presence of a diagnosis code of Stage 3-4 chronic kidney disease (CKD) in those who did not have renal failure. We found 88% of patients who had a diagnosis of Stage 3-4 CKD had eGFR <60 mL/min/1.73m^2, and 90% of those who did not have a diagnosis had eGFR ≥60 mL/min/1.73m^2, which indicates good performance of the diagnosis codes. Moreover, the discrepancy between the diagnosis codes and eGFR could be because some patients may have a temporary decline in eGFR, but later recovered and did not develop to CKD or some patients had serum creatinine tests in facilities that did not submit data to OLDW.

We have also compared the ejection fraction documented in electronic health records and the diagnosis codes of heart failure. Using an ejection fraction cutoff of ≤40% for systolic heart failure diagnosis codes and ejection fraction of ≥50% for diastolic heart failure codes; we observed the specificity of 91% and 81%, respectively and sensitivity of 81% and 91%, respectively. We will include systolic heart failure in the propensity score model as a surrogate for reduced ejection fraction, but we acknowledge the inherent limitations in classification of heart failure by ejection fraction.[33]

We have also conducted validation of the major bleeding diagnosis codes based on the International Society on Thrombosis and Haemostasis (ISTH) criteria[34]: (1) fatal bleeding, and/or, (2) symptomatic bleeding in a critical area or organ, such as intracranial, intraspinal, intraocular, retroperitoneal, intraarticular or pericardial, or intramuscular with compartment

© 2020 Yao X et al. *JAMA Network Open*.

syndrome, and/or, (3) bleeding causing a fall in hemoglobin level of 2 g/dL or more, or leading to transfusion of two or more units of whole blood or red cells. We used ICD-9 and CPT procedure codes to identify transfusion, but we were not able to know the units of whole blood or red cells used in the transfusion. We also identified other procedures to control or manage bleeding, such as endoscopic procedures to address gastrointestinal bleeding, neurosurgical decompression for intracranial bleeding, evacuation of hematoma, or vascular embolization procedures to control bleeding. Among all bleeding events, one in four was bleeding in critical areas, and one third required transfusion. This is generally consistent with previous studies that adapted ISTH definition using administrative data.[35] Nearly 80% of patients had a procedure to control or manage bleeding. In patients with hemoglobin test results, we abstracted the most recent test performed within six months prior to the bleeding. The median time from the previous hemoglobin test to the date of bleeding is 29 (IQR 8-66) days. The median hemoglobin level during the bleeding was 8.2 (IQR 7.3-11.2) g/dL, with a median drop of 2.1 (IQR 1.1-3.6) g/dL. Among patients with transfusion, the median hemoglobin level was 7.3 (IQR 6.5-8.1) g/dL with a median drop of 2.7 (IQR 1.1-3.6) g/dL. In patients without transfusion, the median hemoglobin level was 10.4 (IQR 8.2-12.3) g/dL, with a median drop of 2.1 (IQR 1.2-3.6) g/dL. Overall, 95% of patients identified using diagnosis codes had bleeding in critical area, or a transfusion, or a procedure used to control bleeding, which suggests high specificity of our algorithm. Even in the remaining 5% patients, the hemoglobin level was low, a median of 10.5 (IQR 8.7-12.0), with a median drop of 2.1 (IQR 1.2-3.5) g/dL.

Death will be identified based on the Social Security Death Master File and discharge status. Before November 2011, the Social Security Death Master File has complete mortality data. However, effective on November 1st, 2011, Section 205(r) of the Social Security Act

© 2020 Yao X et al. *JAMA Network Open*.

prohibits the Social Security Administration (SSA) from disclosing state death records that SSA receives through its contracts with the states, except in limited circumstances. Thus, if the SSA knows of a death only from the states and not from any of its other sources of death information, which happens roughly one-third of the time, those death data will not appear on the Death Master File.[36] Using discharge status (i.e. in-hospital death), we typically capture an additional 30% of deaths in addition to what has been captured by Death Master File. Therefore, most of the deaths missing from Death Master File should be captured by discharge status, particularly since most deaths occur in an institutional setting. We acknowledge that a small proportion of patients who died out of hospital and were not captured by Death Master File could be missing, however, this should be non-differential between treatment groups and should not influence our comparison. In fact, the mortality data is more reliable than most measures derived from administrative data, since its specificity is nearly perfect, and the sensitivity is also very high.

5. STATISTICAL METHODS

Based on our prior studies, patients in the early years (e.g., 2007-2010) were younger than those in later years, due to the increasing expansion of Medicare Advantage plans and increasing number of elderly patients in the OLDW database. We will use a weight to adjust for the baseline characteristics. The weight will be calculated from a multinomial logistic regression with the year of the index event as the outcome, and patient characteristics (in the above Table 3) as covariate. The weight will be one divided by the predicted probability of a patient's year of index date.

We will assess the trends of the outcomes in the overall ASCVD population as well as in the three subgroups defined based on a patient's index event: (1) MI, angina, and coronary

© 2020 Yao X et al. *JAMA Network Open*.

revascularization; (2) ischemic stroke and TIA; (3) PAD. We will further assess the trends by age, sex and race/ethnicity. Logistic regression will be used to assess the trends of binary outcomes, including statin use, adherence and statin intolerance. Linear regression will be used to assess the trends of costs. Cox proportional hazards regression will be used to assess MACE, and the cumulative risk of MACE at the end of one year will be calculated using Kaplan –Meier survivor function.

A *P* value less than 0.05 will considered statistically significant for all tests. All tests will be 2-sided. All analyses will be conducted using SAS 9.4 (SAS Institute Inc.) and Stata 14.1 (Stata Corp).

6. LIMITATIONS

Our study relies on administrative data to ascertain baseline characteristics and outcomes, which could be subject to misclassification. However, it is unlikely there is any systematic difference in the ascertainment of comorbidities and outcomes between different treatment groups, and thus, the misclassification should not meaningfully impact our comparisons between drugs. The diagnosis and procedure codes used in this study have been commonly used in previous studies, and demonstrated good performance in our internal validation using linked laboratory results and electronic health records (described in Section 4.4) as well as other validation studies with positive predictive value around 90%.[17, 26, 37-39]

Second, our study will only include privately insured and Medicare Advantage patients. The patient characteristics and outcomes could be different in the Medicaid, Medicare Fee-for-Service, and uninsured populations. However, the insurance coverage rates are high in older Americans. Over 90% of Americans aged 50-64 have health insurance and over 75% had private

© 2020 Yao X et al. *JAMA Network Open*.

health insurance.[40] One in three Medicare patients is enrolled in Medicare Advantage.[41] Although traditionally Medicare Advantage attracted healthier people, after the risk adjustment system was phased in from 2004-2007, the favorable risk selection has been largely reduced.[42]

In fact, the results from this study will be more generalizable than most observational studies using other data sources. Observational studies largely use either administrative data or registries. Some cardiovascular registries focused on cardiology practices for recruitment and patients have to sign informed consent and agree to participate and to be actively followed, and thus the patients in these registries were more selective. Some administrative data are limited within a health system, within a region, or within an age range (e.g., Medicare, Kaiser, etc.). The OptumLabs Data Warehouse contains patients of all ages and races managed at heterogeneous practice settings from all 50 states.[13, 14] The distribution of patient characteristics (e.g., age, sex and race/ethnicity) in the database is similar to those of the general U.S. population.[14] The data are updated monthly and are generally believed to be timely, accurate, and reflective of contemporary practice patterns. The concordance between OptumLabs and everyday practice is a major strength of the data source

© 2020 Yao X et al. *JAMA Network Open*.

eReferences

1. Salami JA, Warraich H, Valero-Elizondo J, Spatz ES, Desai NR, Rana JS, Virani SS, Blankstein R, Khera A, Blaha MJ, Blumenthal RS, Lloyd-Jones D and Nasir K. National Trends in Statin Use and Expenditures in the US Adult Population From 2002 to 2013: Insights From the Medical Expenditure Panel Survey. *JAMA cardiology*. 2017;2:56-65.

2. Benjamin EJ, Muntner P, Alonso A, Bittencourt MS, Callaway CW, Carson AP, Chamberlain AM, Chang AR, Cheng S, Das SR, Delling FN, Djousse L, Elkind MSV, Ferguson JF, Fornage M, Jordan LC, Khan SS, Kissela BM, Knutson KL, Kwan TW, Lackland DT, Lewis TT, Lichtman JH, Longenecker CT, Loop MS, Lutsey PL, Martin SS, Matsushita K, Moran AE, Mussolino ME, O'Flaherty M, Pandey A, Perak AM, Rosamond WD, Roth GA, Sampson UKA, Satou GM, Schroeder EB, Shah SH, Spartano NL, Stokes A, Tirschwell DL, Tsao CW, Turakhia MP, VanWagner LB, Wilkins JT, Wong SS and Virani SS. Heart Disease and Stroke Statistics-2019 Update: A Report From the American Heart Association. *Circulation*. 2019;139:e56-e66.

3. Baigent C, Blackwell L, Emberson J, Holland LE, Reith C, Bhala N, Peto R, Barnes EH, Keech A, Simes J and Collins R. Efficacy and safety of more intensive lowering of LDL cholesterol: a meta-analysis of data from 170,000 participants in 26 randomised trials. *Lancet (London, England)*. 2010;376:1670-81.

4. Shah ND, Dunlay SM, Ting HH, Montori VM, Thomas RJ, Wagie AE and Roger VL. Long-term medication adherence after myocardial infarction: experience of a community. *Am J Med*. 2009;122:961.e7-13.

© 2020 Yao X et al. *JAMA Network Open*.

5. Chowdhury R, Khan H, Heydon E, Shroufi A, Fahimi S, Moore C, Stricker B, Mendis S, Hofman A and Mant J. Adherence to cardiovascular therapy: a meta-analysis of prevalence and clinical consequences. *European heart journal*. 2013;34:2940-2948.

6. Rasmussen JN, Chong A and Alter DA. Relationship between adherence to evidence-based pharmacotherapy and long-term mortality after acute myocardial infarction. *Jama*. 2007;297:177-186.

7. De Vera MA, Bhole V, Burns LC and Lacaille D. Impact of statin adherence on cardiovascular disease and mortality outcomes: a systematic review. *British journal of clinical pharmacology*. 2014;78:684-698.

8. Rodriguez F, Maron DJ, Knowles JW, Virani SS, Lin S and Heidenreich PA. Association of Statin Adherence With Mortality in Patients With Atherosclerotic Cardiovascular Disease. *JAMA cardiology*. 2019;4:206-213.

9. van Driel ML, Morledge MD, Ulep R, Shaffer JP, Davies P and Deichmann R. Interventions to improve adherence to lipid-lowering medication. *The Cochrane database of systematic reviews*. 2016;12:Cd004371.

10. Stone NJ, Robinson JG, Lichtenstein AH, Merz CNB, Blum CB, Eckel RH, Goldberg AC, Gordon D, Levy D and Lloyd-Jones DM. 2013 ACC/AHA guideline on the treatment of blood cholesterol to reduce atherosclerotic cardiovascular risk in adults: a report of the American

© 2020 Yao X et al. *JAMA Network Open*.

College of Cardiology/American Heart Association Task Force on Practice Guidelines. *Journal of the American College of Cardiology*. 2014;63:2889-2934.

11. Korhonen MJ, Robinson JG, Annis IE, Hickson RP, Bell JS, Hartikainen J and Fang G. Adherence Tradeoff to Multiple Preventive Therapies and All-Cause Mortality After Acute Myocardial Infarction. *J Am Coll Cardiol*. 2017;70:1543-1554.

12. Rosenson RS, Farkouh ME, Mefford M, Bittner V, Brown TM, Taylor B, Monda KL, Zhao H, Dai Y and Muntner P. Trends in Use of High-Intensity Statin Therapy After Myocardial Infarction, 2011 to 2014. *J Am Coll Cardiol*. 2017;69:2696-2706.

13. Wallace PJ, Shah ND, Dennen T, Bleicher PA and Crown WH. Optum Labs: building a novel node in the learning health care system. *Health affairs (Project Hope)*. 2014;33:1187-1194.

14. Optum. Optum Research Data Assets. 2015.

15. Yao X, Gersh BJ, Holmes DR, Jr and et al. Association of surgical left atrial appendage occlusion with subsequent stroke and mortality among patients undergoing cardiac surgery. *Jama*. 2018;319:2116-2126.

16. Kernan WN, Ovbiagele B, Black HR, Bravata DM, Chimowitz MI, Ezekowitz MD, Fang MC, Fisher M, Furie KL, Heck DV, Johnston SC, Kasner SE, Kittner SJ, Mitchell PH, Rich MW, Richardson D, Schwamm LH and Wilson JA. Guidelines for the prevention of stroke in

© 2020 Yao X et al. *JAMA Network Open*.

patients with stroke and transient ischemic attack: a guideline for healthcare professionals from the American Heart Association/American Stroke Association. *Stroke*. 2014;45:2160-236.

17. Fan J, Arruda-Olson AM, Leibson CL, Smith C, Liu G, Bailey KR and Kullo IJ. Billing code algorithms to identify cases of peripheral artery disease from administrative data. *Journal of the American Medical Informatics Association : JAMIA*. 2013;20:e349-54.

18. Hershman DL, Tsui J, Wright JD, Coromilas EJ, Tsai WY and Neugut AI. Household net worth, racial disparities, and hormonal therapy adherence among women with early-stage breast cancer. *Journal of Clinical Oncology*. 2015;33:1053-1059.

19. National Quality Measures. Comprehensive diabetes care: percentage of members 18 to 75 years of age with diabetes (type 1 and type 2) whose most recent blood pressure reading is less than 140/90 mm Hg (controlled). 2015.

20. Zhou L, Stearns SC, Thudium EM, Alburikan KA and Rodgers JE. Assessing Medicare Part D claim completeness using medication self-reports: the role of veteran status and Generic Drug Discount Programs. *Medical care*. 2015;53:463-70.

21. Stuart B and Loh EF. Medicare Part D Enrollees’ Use of Out-of-Plan Discounted Generic Drugs. *Journal of the American Geriatrics Society*. 2012;60:387-388.

© 2020 Yao X et al. *JAMA Network Open*.

22. Dunn A, Grosse SD and Zuvekas SH. Adjusting Health Expenditures for Inflation: A Review of Measures for Health Services Research in the United States. *Health services research*. 2018;53:175-196.

23. Agency for Healthcare Research and Quality. Using appropriate price indices for analyses of health care expenditures or income across multiple years. 2018.

24. Serban MC, Colantonio LD, Manthripragada AD, Monda KL, Bittner VA, Banach M, Chen L, Huang L, Dent R, Kent ST, Muntner P and Rosenson RS. Statin Intolerance and Risk of Coronary Heart Events and All-Cause Mortality Following Myocardial Infarction. *J Am Coll Cardiol*. 2017;69:1386-1395.

25. Colantonio LD, Kent ST, Huang L, Chen L, Monda KL, Serban MC, Manthripragada A, Kilgore ML, Rosenson RS and Muntner P. Algorithms to Identify Statin Intolerance in Medicare Administrative Claim Data. *Cardiovasc Drugs Ther*. 2016;30:525-533.

26. Tirschwell DL and Longstreth W. Validating administrative data in stroke research. *Stroke*. 2002;33:2465-2470.

27. Cunningham A, Stein CM, Chung CP, Daugherty JR, Smalley WE and Ray WA. An automated database case definition for serious bleeding related to oral anticoagulant use. *Pharmacoepidemiology and drug safety*. 2011;20:560-566.

© 2020 Yao X et al. *JAMA Network Open*.

28. Arnason T, Wells P, Van Walraven C and Forster A. Accuracy of coding for possible warfarin complications in hospital discharge abstracts. *Thrombosis research*. 2006;118:253-262.

29. Hwang YJ, Shariff SZ, Gandhi S, Wald R, Clark E, Fleet JL and Garg AX. Validity of the International Classification of Diseases, Tenth Revision code for acute kidney injury in elderly patients at presentation to the emergency department and at hospital admission. *BMJ open*. 2012;2:e001821.

30. Wilchesky M, Tamblyn RM and Huang A. Validation of diagnostic codes within medical services claims. *Journal of clinical epidemiology*. 2004;57:131-141.

31. Yao X, Shah ND, Sangaralingham LR, Gersh BJ and Noseworthy PA. Non-vitamin K antagonist oral anticoagulant dosing in patients with atrial fibrillation and renal dysfunction. *J Am Coll Cardiol*. 2017;69:2779-2790.

32. Yao X, Tangri N, Gersh BJ, Sangaralingham LR, Shah ND, Nath KA and Noseworthy PA. Renal Outcomes in Anticoagulated Patients With Atrial Fibrillation. *J Am Coll Cardiol*. 2017;70:2621-2632.

33. Shah KS, Xu H, Matsouaka RA, Bhatt DL, Heidenreich PA, Hernandez AF, Devore AD, Yancy CW and Fonarow GC. Heart Failure With Preserved, Borderline, and Reduced Ejection Fraction: 5-Year Outcomes. *J Am Coll Cardiol*. 2017.

© 2020 Yao X et al. *JAMA Network Open*.

34. Schulman S and Kearon C. Definition of major bleeding in clinical investigations of antihemostatic medicinal products in non-surgical patients. *Journal of Thrombosis and Haemostasis*. 2005;3:692-694.

35. Jasuja GK, Reisman JI, Miller DR, Berlowitz DR, Hylek EM, Ash AS, Ozonoff A, Zhao S and Rose AJ. Identifying major hemorrhage with automated data: results of the Veterans Affairs study to improve anticoagulation (VARIA). *Thrombosis research*. 2013;131:31-36.

36. da Graca B, Filardo G and Nicewander D. Consequences for healthcare quality and research of the exclusion of records from the death master file. *Circulation: Cardiovascular Quality and Outcomes*. 2013;6:124-128.

37. Kumamaru H, Judd SE, Curtis JR, Ramachandran R, Hardy NC, Rhodes JD, Safford MM, Kissela BM, Howard G and Jalbert JJ. Validity of claims-based stroke algorithms in contemporary medicare data reasons for geographic and racial differences in stroke (REGARDS) study linked with medicare claims. *Circulation: Cardiovascular Quality and Outcomes*. 2014;7:611-619.

38. Kokotailo RA and Hill MD. Coding of stroke and stroke risk factors using international classification of diseases, revisions 9 and 10. *Stroke*. 2005;36:1776-1781.

39. Jensen PN, Johnson K, Floyd J, Heckbert SR, Carnahan R and Dublin S. Identifying atrial fibrillation from electronic medical data: a systematic review. *Pharmacoepidemiology and drug safety*. 2012;21:141-147.

© 2020 Yao X et al. *JAMA Network Open*.

40. Barnett JC and Vornovitsky MS. Health insurance coverage in the United States: 2015. *US Census Bureau, Current Population Reports, Report*. 2016.

41. Jacobson G, Casillas G, Damico A, Neuman T and Gold M. Medicare Advantage 2016 spotlight: enrollment market update. 2016.

42. McWilliams JM, Hsu J and Newhouse JP. New risk-adjustment system was associated with reduced favorable selection in medicare advantage. *Health affairs (Project Hope)*. 2012;31:2630-40.

© 2020 Yao X et al. *JAMA Network Open*.
